# Integrative QTL Identification, Fine Mapping and Candidate Gene Analysis of a Major Locus *qLTG3a* for Seed Low-Temperature Germinability in Rice

**DOI:** 10.1186/s12284-021-00544-2

**Published:** 2021-12-15

**Authors:** Zhaoyuan Pan, Bin Tan, Guiyuan Cao, Rongqi Zheng, Meng Liu, Ruizhen Zeng, Shaokui Wang, Haitao Zhu, Heng Ye, Guangmiao Zhao, Wei Cao, Guifu Liu, Guiquan Zhang, Yuliang Zhou

**Affiliations:** 1 Guangdong Key Laboratory of Plant Molecular Breeding and State Key Laboratory for Conservation and Utilization of Subtropical Agro-Bioresources, South China Agricultural University, Guangzhou, 510642 China; 2 Division of Plant Sciences, University of Missouri, Columbia, MO 65211 USA

**Keywords:** Substitution mapping, Bulk segregant analysis, Low temperature, Seed germination vigor, Candidate gene

## Abstract

Low-temperature germinability (LTG) is an important agronomic trait that can affect the planting time, planting area, and grain yield of staple crops, such as rice. However, the genetic mechanism of LTG is still unclear. In this study, a multi-parental permanent population with 208 single segment substitution lines (SSSLs) was used to conduct a genetic dissection for LTG across four cropping seasons. LTG was a typical quantitative trait with a high combined broad-sense heritability of 0.71. By comparison with the recipient parent, Huajingxian74, 24 SSSLs were identified as carrying LTG QTLs, which were further merged into integrated QTLs with shorter genetic distances by substitution mapping. Finally, 14 LTG QTLs were mapped on ten chromosomes, including seven positive-effect and seven negative-effect QTLs, with additive effect contributions ranging from 19.2 to 39.9%. *qLTG3a*, a main-effect and novel QTL, was confirmed by bulk segregant analysis using an F_2_ segregating population, and five key recombinants were selected to develop F_3_ populations for progeny testing. Marker-trait association analysis fine mapped *qLTG3a* to a 332.7-kb physical region between markers M6026 and M6341. Within this interval, 40 annotated genes were revealed, and three genes (*Os03g0213300*, *Os03g0214400*, and *Os03g0214600*) were considered as pivotal candidate genes for *qLTG3a* based on their sequence variations and expression patterns. Besides low temperature, *qLTG3a* can also enhance seed germination under standard temperature and osmotic stress. In summary, this study identified some genetic factors regulating LTG and opened a new window for breeding elite direct-seeded rice varieties. It will help reduce the climate risk in the production process of rice, which is of great significance to ensuring food security.

**Supplementary Information:**

The online version contains supplementary material available at 10.1186/s12284-021-00544-2.

## Introduction

Seed germination, a critical step in the plant life cycle, is a complex process coordinately regulated by internal genetic factors and external environmental cues (Ma et al. 2017; Rajjou et al. 2012). Low temperature is one of the principal restriction factors that limit crop growth and development (Ding et al. 2020). As a thermophilic crop, rice (*Oryza sativa *L.) is sensitive to chilling stress through its life cycle, especially during the germination stage. However, environmental temperature is frequently below 15 °C in high-latitude and high-altitude rice cultivation regions or early spring seasons, causing a severe rice yield reduction due to low germination rates, delayed seedling emergence, and abnormal seedlings (Liu et al. 2018). Recently, direct seeding cultivation techniques were widely adopted in Asian countries, which increased the risk of encountering cold stress during germination (Mahender et al. 2015; Wang et al. 2018a). High low-temperature germinability (LTG) of rice varieties can extend rice planting areas and growing season to meet the expanding food demand. Therefore, it is of considerable significance to fully understand the genetic basis of LTG in rice.

LTG is a complex quantitative trait controlled by multiple genes. In the past 20 years, researchers have identified more than 100 QTLs for LTG on the 12 rice chromosomes using bi-parental mapping methods based on phenotypic traits such as germination percentage and germination index (Chen et al. 2006; Fujino et al. 2004; Ji et al. 2009; Jiang et al. 2006, 2017; Miura et al. 2001; Wang et al. 2011; Xie et al. 2014). Besides, as an alternative strategy, genome-wide association study (GWAS) has increasingly applied for LTG genetic research to dissect the variation of natural rice accessions (Fujino et al. 2015; Sales et al. 2017; Wang et al. 2018b; Yang et al. 2020).

QTL analysis lays the foundation for fine mapping and map-based cloning of some major LTG QTLs. For example, *qLTG-9* was fine mapped using a near-isogenic line population between markers L9-25D and ID-1 to a 72.3-kb physical region on chromosome 9 containing five candidate genes (Li et al. 2013). *qSV-5c*, a major QTL of radical length and germination rate under optimal and low temperature, was located between SNP3 and CAPS2, a genomic region of approximately 400 kb on chromosome 1 (Xie et al. 2014). By progeny test, *qLTG6* was delimited to a 45.8-kb physical interval between M002 and M008, and *LOC_Os06g01320* was considered as a possible target gene (Jiang et al. 2020). Currently, only two LTG-related genes, *qLTG3-1* and *OsSAP16*, have been cloned in rice. *qLTG3-1* (*Os03g0103300*) encoded a glycine-rich protein of unknown function and was strongly expressed in the embryo during seed germination (Fujino et al. 2008). Further genome-wide analysis of gene expression and regulation revealed that *qLTG3-1* might induce programmed cell death to weaken the tissues covering the embryo and reduce the mechanical resistance of the coleoptile (Fujino and Matsuda 2010; Fujino et al. 2008). *OsSAP16* was identified through GWAS; it encodes a stress-associated protein containing two AN1-C2H2 zinc finger domains and acts as an essential LTG regulator (Wang et al. 2018c). These findings of LTG genes have brought some breakthroughs in the genetic control of LTG in rice. However, extra efforts are needed to enrich our knowledge about this critical agronomical trait's genetic and molecular mechanisms.

Single segment substitution lines (SSSLs) contain a single homozygous marker-defined chromosome segment from a donor parent in a recurrent parent background (Zhang et al. 2004). Unlike traditional segregated populations, SSSLs, a permanent advanced mapping population, is a powerful tool for QTL mapping and gene cloning because it can eliminate the interference of genetic background and improve the accuracy of QTL mapping. Recent studies have demonstrated that SSSLs play a crucial role in understanding the genetic mechanisms of important agronomic traits such as seed dormancy, grain shape, and heading date in rice (Wang et al. 2012; Zhou et al. 2017; Zhu et al. 2018).

In this study, the genetic dissection of rice LTG trait was carried out using 208 SSSLs with abundant genetic diversity conferred by 24 donor cultivars in the genetic background of Huajingxian 74 (HJX74) (Li et al. 2021), an elite *indica* recipient parent, in four cropping seasons. The objects of this research were (1) to identify QTLs controlling LTG using germination percentage at 15 °C as phenotypic data in a rice SSSL population; (2) to integrate and narrow down the QTLs located at similar chromosome locations by substitution mapping; and (3) to validate and fine map a novel major QTL, *qLTG3a*, and predict the possible candidate genes by sequence and expression analysis. These works will provide new insights into the genetic architecture of LTG and establish the basis for map-based cloning of the casual gene underline *qLTG3a*.

## Materials and Methods

### Plant Materials

Genetic analysis of LTG was conducted using 208 SSSLs, which were selected from a 1529 SSSLs library constructed with HJX74 as the recipient and 24 varieties from different countries as donors (Xi et al. 2006; Zhang et al. 2004). Each SSSL contained only one substituted segment from 24 donors (including 14 *Indica* and 10 *japonica* varieties) on the 12 chromosomes of rice in the genetic background of HJX74 (Additional file 1: Table S1). The length of substituted segments was 0.4–70.0 cM, the average length was 15.84 cM, and the genome coverage was 68.0% (Additional file 1: Fig. S1).

### Field Planting

Rice plants were grown in an experimental field in South China Agricultural University, Guangzhou, China (at approximately 113° E and approximately 23° N). Each SSSL was grown in one block consisting of two rows of 20 plants in 2015E, 2015L, 2016E, or 2016L. "E" represents the early cropping season from March to July, and "L" represents the late cropping season from July to November. Depending on the number of SSSLs, the control line HJX74 was grown in several blocks, and the differences among these blocks were evaluated by one-way ANOVA to assess the effects of the field environment. All rice materials were grown in a wide-narrow row method, 33.33 cm for the wide row and 16.67 cm for the narrow row, with a completely randomized design and regular management. One to two weeks after transplanting to the paddy field, the fresh leaves of rice seedlings were collected and used for genomic DNA extraction. Marker genotyping was conducted with SSR markers to detect the substituted segment of each SSSL in every growing season.

### Phenotypic Evaluation of LTG

LTG was evaluated as previously described by Cao et al. (2017) with the following changes. Plants from the middle of each block were tagged for heading date when the first panicle emerged. About 32–35 days after heading, the main panicles of three to five individual plants for each SSSL line were harvested according to the seed maturity status. After threshing and cleaning, the seeds were dried in an oven at 50 °C for seven days to break seed dormancy. About 100–200 well-developed seeds of each plant were randomly distributed into two 9-cm Petri-dishes with 10 ml distilled water per dish. The petri-dishes were then transferred to several large sealed plastic boxes (34.7 × 24.5 × 12.5 cm) with two layers of wet filter paper on the bottom and finally placed in a 15 °C chamber in the dark for ten days. A seed with radicle length longer than 1 mm in 2015E, 2015L, and 2016L, or 2 mm in 2016E, was counted as germinated from the 4th to 10th day after sowing (He et al. 2019). The germination percentages of the 7th day (2015E, 2015L, and 2016L) and 10th day (2016E) showed great variations in the SSSLs and were accordingly used as the phenotypic data of LTG. After germination at 15 °C, the seeds with germination percentages less than 80% were moved into a chamber of 30 °C to eliminate the secondary dormancy induced by low temperature.

### QTL Analysis and Substitution Mapping

QTL analysis was performed following the method as previously described (Eshed and Zamir 1995; Zhou et al. 2017). To normalize the variance, the arcsine-transformed germination data were used in the Student's *t* tests to calculate significant differences between SSSLs and HJX74. When the germination percentage's *P valu*e was less than 0.001 in at least two cropping seasons, a putative LTG QTL was considered on the substituted segment of the corresponding SSSL. The nomenclature of identified QTLs follows the general rules in rice. A QTL's name consists of three parts: the abbreviation of the trait, the chromosome number, and the QTL number, which is sequenced from the short arm end to the long arm end of the chromosome. Assuming that each SSSL carried only one QTL, the additive effect (*a*) of a putative QTL accounted for half of the phenotypic difference in germination percentages between an SSSL and HJX74; the percentage of additive effect contribution (*R*^*2*^) was calculated as the additive effect divided by the mean phenotypic value of HJX74. If a QTL was simultaneously detected in multiple SSSLs with overlapping segments, then substitution mapping method was applied to reduce the region of a QTL as previously described by Wissuwa et al. (2002) and Zhou et al. (2017).

### Bulk Segregant Analysis (BSA) Sequencing

In this study, a major QTL, *qLTG3a*, was simultaneously detected on the overlapping substituted segments of S5, S6, and S7 on chromosome 3. S6 contains a segment derived from Katy, a *japonica* donor parent, and was selected as a representative of *qLTG3a* in further analysis. For validation of *qLTG3a*, S6 was backcrossed with the *indica* recurrent parent HJX74 to produce an F_2_ segregating population with 356 individual plants. Young leaves of the 356 F_2_ individuals were collected separately and stored at -80℃. After evaluation of LTG, the same size leave samples of 30 extremely low-temperature-tolerant and 30 extremely low-temperature-sensitive plants were bulked as LTG-pool and WT-pool, respectively. Total genomic DNA of segregation pools was extracted using the CTAB method. DNA quality was determined by Nanodrop (Thermo Fisher Scientific, Waltham, MA). The two DNA pools, parental cultivars S6 and HJX74 were sequenced with coverage of 30X using Illumina sequencing strategy at Genedenovo (Guangzhou, China). Filtered reads were compared with the R498 (Shuhui498, an *indica* variety) genome sequence to identify single-nucleotide polymorphisms (SNPs) and Insertion-deletions (InDels) using Burrows-Wheeler Aligner (BWA, v0.7.12). By calculating SNP-index and ΔSNP-index, the distribution of ΔSNP-index was fitted on 12 chromosomes. The 95% confidence level was selected as the threshold to determine the linkage interval. Also, ΔInDel-index was calculated for subsequent sequence variation analysis of candidate genes.

### Marker Development and Fine Mapping

New high-density markers were designed on the substituted segment of S6 flanked by RM231 and RM563 based on InDel variations identified by BSA sequencing and the difference between the Nipponbare and 9311 genome sequences. Fourteen polymorphic InDel markers were used for genotyping the 356 F_2_ individual plants to conduct frequency distribution analysis and select recombinants for the *qLTG3a* locus. Based on the marker genotypes, five recombinants, named S6_R1 to S6_R5, were detected, and more than 70 progeny lines for each recombinant were genotyped with a marker on the heterozygous region of *qLTG3a* and used to evaluate LTG. The data of genotype and phenotype were employed in a mark-trait association analysis to fine map *qLTG3a*. The primer sequence used in fine mapping can be found in supplementary data (Additional file 1: Table S2).

### Marker-Trait Association Analysis and Estimation of Genetic Effects

The marker-trait association and genetic effects of *qLTG3a* on LTG were examined using the SAS program (SAS Institute 2011), as previously reported by Ye et al. (2015). Data from the recombinant-derived progeny populations were analyzed by line correlation analysis to determine the mark-trait associations at the *qLTG3a* locus. Genotypes for a marker locus were coded as 1, 2, and 3 for HJX74-like homozygote, heterozygote, and S6-like homozygote, respectively, to correlate with the trait value for LTG. Additive and dominance effects of *qLTG3a* on LTG were assessed using the linear regression model 1: *y*_*ij*_ = *μ* + *αx* + *dz* + *ε*_*ij*_, where *y*_*ij*_ is the phenotypic value for the *j*th plant of the *i*th marker genotype; *μ* is the model mean; *x* is the dummy variable for the additive component and was coded as -1, 0, and 1 when *i* = 1, 2, and 3, respectively; *z* is the dummy variable for the dominance component and was coded as 0, 1, or 0 when *i* = 1, 2, or 3; *a* and *d* are regression coefficients that estimate the additive and dominance effects, respectively; and *ε*_*ij*_ is the error term of the model.

### Candidate Gene Identification

Fine mapping and BSA-seq data were comprehensively used to dig the candidate genes underlying *qLTG3a*. Firstly, the possible candidate genes in the QTL region were chosen according to the functional annotations from the Rice Annotation Project Database (RAP-DB, https://rapdb.dna.affrc.go.jp). Then, based on the whole genome resequencing data by BSA-seq, we further focused on those candidate genes with nonsynonymous SNPs, or InDel mutations in the upstream and exonic regions between the two parents, S6 and HJX74. Finally, those genes that are abundantly or predominantly expressed in seeds were considered as key candidate genes.

### RNA Extraction and qRT-PCR Analysis

To verify the temporal and spatial expression patterns of candidate genes, qRT-PCR was conducted with different rice organs of HJX74, including developing seeds at 20 and 30 DAF, mature roots, stems and leaves, and 3-day-old seedlings. Total RNA was isolated from the above tissues by using the RaPure Plant RNA Kit (Magen) according to the manufacturer’s instructions. First-strand cDNA was synthesized from the total RNA with the *Evo M-MLV* RT Kit with gDNA Clean for qPCR (Accurate biology). qRT-PCR was performed on a CFX 96 (Bio-Rad) in a 20 μl system containing 2 μl of cDNA, 0.4 μl of gene-specific primers (10 μM), 10 μl of SYBR Green *Pro Taq* HS Premix (Accurate biology), and 7.2 μl of RNase-free water according to the manufacturer’s protocol. The PCR program was 95 °C denaturation for 30 s followed by 40 cycles of 95 °C for 5 s and 60 °C for 30 s. The specificity of the PCR amplification was checked with dissociation curves (65–95 °C) following the final cycle of the PCR. *OsActin1* (*Os03g0718100*) was used as an internal reference for standardization of cDNA amounts. Primers for qRT-PCR are listed in Additional file 1: Table S2. Data represent three biological replicates and three technical replicates.

### Germination Assays

To investigate the role of *qLTG3a* responding to other stresses, the germination performances of S6 and HJX74 were analyzed in various environmental stresses. Each treatment was biologically repeated three times. About 50 seeds for each replicate were placed in 9-cm Petri dishes and cultured under normal temperature (30 °C), high temperature (38 °C), osmotic stress (3% mannitol, 30 °C), and high-salt stress (200 mM NaCl, 30 °C). The germinated seeds were counted daily for one to seven days, depending on germination profiles under different stress conditions.

## Results

### Phenotypic Variation of LTG in SSSLs

**Fig. 1:**
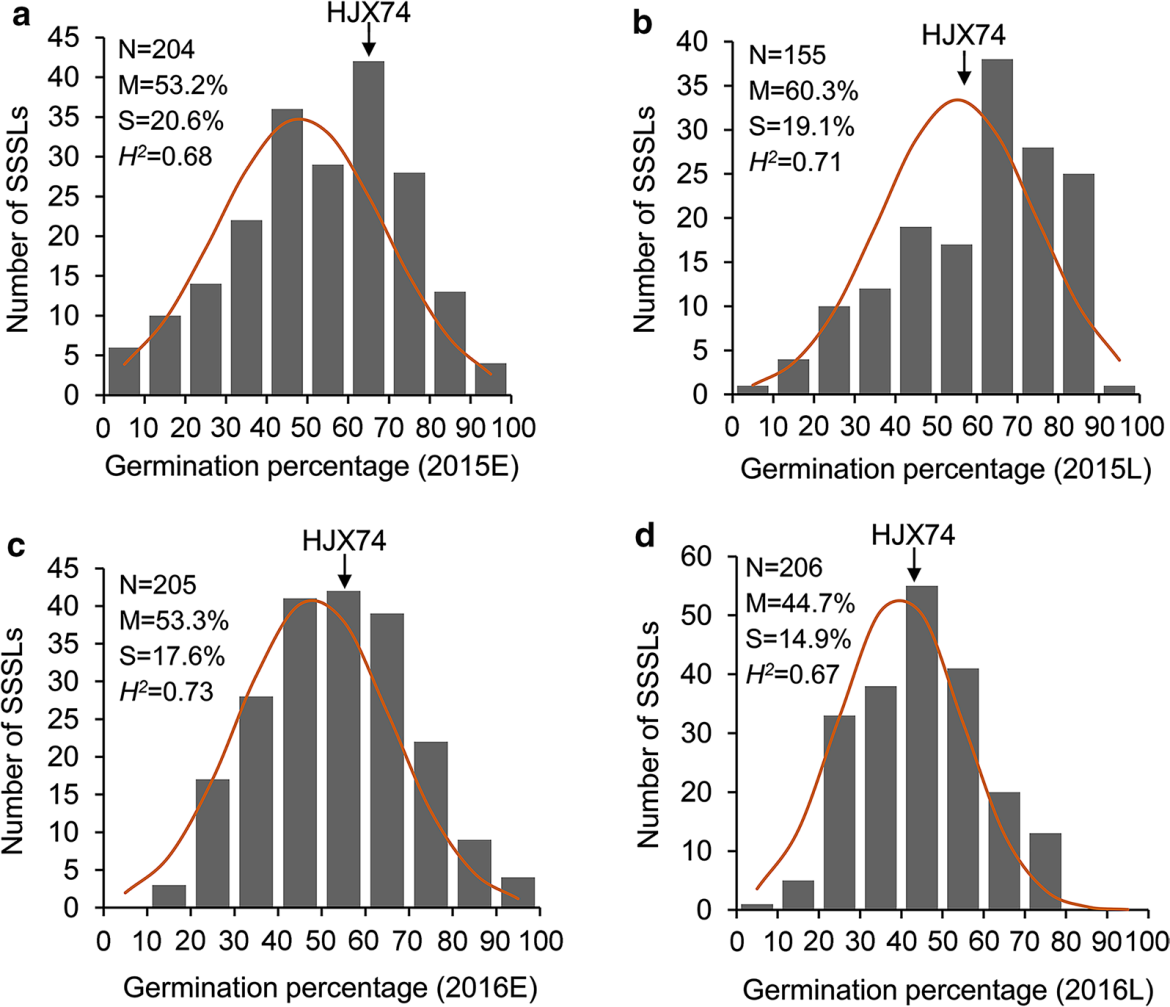
Phenotypic distribution of LTG for 208 rice SSSLs in four cropping seasons. **a** 2015E, **b** 2015L, **c** 2016E, **d** 2016L. Parameters are the number of SSSLs (N) and mean (M) and standard deviation (S) for germination of the freshly harvested seeds at 15 °C. The arrow indicates the germination percentage of the recipient parent HJX74. Broad-sense heritability (*H*^2^) of LTG was estimated by the germination rates of SSSLs in each cropping season. 2015E, 2015L, 2016E, and 2016L represent the early (E) and late (L) cropping seasons in 2015 and 2016, respectively

The germination percentages at 15 °C of HJX74 and 208 SSSLs were investigated to evaluate the phenotypic variation of LTG in four growing seasons from 2015 to 2016. The recipient parent HJX74 was planted in 10, 9, 9, and 11 blocks, and the average LTG value was 67.7 ± 1.4, 57.4 ± 1.3, 55.3 ± 1.4, and 44.1 ± 1.2% in 2015E, 2015L, 2016E, and 2016L, respectively (Fig. 1). In addition, the mean LTG values of HJX74 in different blocks of each cropping season were analyzed by one-way ANOVA in 2015 and 2016. The results showed no significant difference in each cropping season, indicating the field environment among different blocks was relatively consistent (Additional file 1: Table S3). On the other hand, the LTG values in SSSLs ranged from 2.8 to 98.1, 9.8–90.8, 12.7–93.4 and 8.7–79.3%, with an average of 53.2, 60.3, 53.3 and 44.7% in 2015E, 2015L, 2016E and 2016L, respectively (Fig. 1). Our data proved that LTG is a typical quantitative trait, as the low-temperature germination percentages in SSSLs displayed wide variations and normal distributions in multiple environments. As shown in Fig. 1, the broad-sense heritability (*H*^*2*^) of LTG was 0.68, 0.71, 0.73, and 0.67 in 2015E, 2015L, 2016E, and 2016L, respectively; the combined broad-sense heritability was 0.71 across four environments. These data indicated that genetic factors mainly contributed to the phenotypic variations observed in SSSLs.

### Identification of SSSLs with Putative LTG QTLs

For QTL analysis, the LTG values of each SSSL and the recipient parent HJX74 were analyzed by Student's *t* test. The results demonstrated that 24 SSSLs displayed significantly different phenotypic values in comparison to HJX74 (*P* < 0.001), implying that these SSSLs carry putative LTG QTLs on their corresponding substituted segments (Additional file 1: Fig. S2; Table S4).

As shown in Table 1, the 24 SSSLs originated from 10 donor parents, including Tetep (W01), Zihui 100 (W05), Katy (W06), IR64 (W08), Basmati 385 (W09), Basmati 370 (W11), Lianjian33 (W14), Ganxiangnuo (W17), Lemont (W23), and IAPAR9 (W27), of which Katy contributed 12 or half of the SSSLs. The substituted segments of the 24 SSSLs were distributed on ten chromosomes except 10 and 12, and their lengths ranged from 6.5 to 54.5 cM (Table 1). It should be pointed out that some of the 24 SSSLs shared overlapping segments, and they were therefore considered to contain the same QTL of different alleles corresponding to their donor parents. Accordingly, seven positive-effect QTLs, *qLTG2*, *qLTG3a*, *qLTG5*, *qLTG6*, *qLTG8a*, *qLTG8b,* and *qLTG11a*, were identified on the substituted segments of 14 SSSLs, with additive effects ranging from 0.06 to 0.18 and additive effect contributions ranging from 8.9 to 36.2% (Table 1). In contrast, seven negative-effect QTLs, *qLTG1*, *qLTG3b*, *qLTG4*, *qLTG7a*, *qLTG7b*, *qLTG9, and qLTG11b*, were detected on the substituted segments of 10 SSSLs. The additive effects were between − 0.10 and − 0.24, and additive effect contributions were between 19.2 and 39.9% (Table 1).

**Table 1:** Summary of the SSSLs detected with putative LTG QTLs in four field environments

SSSL code	Donor code	Chr	Substituted segment^a^	Length (cM)	Putative QTL	a*	*R*^*2*^ (%)*	Cropping season^b^	Detected season^c^
S1	W14	1	RM246–RM297-RM212–RM315	16.7	*qLTG1*	− 0.18 ± 0.02	28.6 ± 7.0	abcd	ab
S2	W14	1	RM5–RM237-RM212–RM315	34.2	*qLTG1*	− 0.17 ± 0.10	29.4 ± 11.8	abcd	acd
S3	W09	2	RM106–RM263-RM530–RM240	30.2	*qLTG2*	0.10 ± 0.04	20.0 ± 4.5	abcd	bd
S4	W08	2	PSM122–RM263-RM166–RM213	54.5	*qLTG2*	0.11 ± 0.06	18.9 ± 10.9	abcd	bc
S5	W06	3	RM175–RM489-PSM428–PSM429	16.1	*qLTG3a*	0.16 ± 0.02	33.0 ± 1.0	acd	cd
S6	W06	3	RM231–RM489-RM218–RM563	28.7	*qLTG3a*	0.16 ± 0.01	29.8 ± 7.8	acd	acd
S7	W06	3	RM489–RM545-RM218–RM563	21.9	*qLTG3a*	0.18 ± 0.01	36.2 ± 5.4	acd	cd
S8	W17	3	PSM127–RM426-RM168–RM186	16.4	*qLTG3b*	− 0.10 ± 0.01	19.2 ± 5.8	abcd	ad
S9	W06	4	RM119–RM273-RM252–RM241	12.9	*qLTG4*	− 0.14 ± 0.01	25.8 ± 7.0	acd	ad
S10	W06	4	PSM194–RM273-RM252–RM241	16.2	*qLTG4*	− 0.16 ± 0.08	26.9 ± 6.6	acd	ad
S11	W06	4	PSM358–PSM196-RM252–RM241	47.0	*qLTG4*	− 0.20 ± 0.13	32.9 ± 13.8	acd	ad
S12	W11	5	RM480–RM31-PSM386–End	8.4	*qLTG5*	0.15 ± 0.02	28.0 ± 1.5	abcd	bcd
S13	W01	5	PSM384–RM178-RM334–End	22.4	*qLTG5*	0.14 ± 0.01	28.7 ± 2.2	bcd	cd
S14	W27	6	End–RM508-RM217–RM253	16.6	*qLTG6*	0.06 ± 0.01	8.9 ± 0.5	acd	ac
S15	W23	6	End–RM508-RM121–RM136	44.9	*qLTG6*	0.15 ± 0.02	29.9 ± 9.9	abcd	bd
S16	W06	7	RM481–PSM142–PSM143	10.0	*qLTG7a*	− 0.22 ± 0.09	39.4 ± 3.7	acd	ad
S17	W05	7	PSM142–RM2-RM10–PSM353	45.4	*qLTG7b*	− 0.24 ± 0.08	39.9 ± 8.9	abcd	abc
S18	W06	8	PSM155–RM547–PSM393	6.5	*qLTG8a*	0.15 ± 0.02	26.9 ± 4.4	abcd	bc
S19	W06	8	RM284–RM556-RM210–RM80	9.1	*qLTG8b*	0.17 ± 0.03	33.6 ± 1.3	abcd	cd
S20	W06	8	PSM394–RM515-OSR7–RM447	36.4	*qLTG8b*	0.15 ± 0.01	28.3 ± 2.4	abcd	bcd
S21	W27	9	RM105–RM434-RM410–RM257	17.1	*qLTG9*	− 0.21 ± 0.01	34.2 ± 6.0	abcd	ac
S22	W06	11	PSM409–PSM412–PSM414	28.8	*qLTG11a*	0.16 ± 0.01	32.2 ± 4.3	abcd	bd
S23	W06	11	PSM174–PSM175-RM536–PSM415	40.2	*qLTG11a*	0.15 ± 0.03	30.3 ± 9.8	abcd	cd
S24	W17	11	RM21–PSM365-PSM417–PSM460	15.4	*qLTG11b*	− 0.19 ± 0.07	34.1 ± 9.7	acd	acd

*a = average additive effect of the QTL in different detected seasons; *R*^2^ (%) = average additive effect contribution (%) of the QTL in different detected seasons

^a^The single lines in the middle of markers indicate substituted segments while the double lines on the border of markers indicate segments recombination might appear. SSR markers labeled "RM" were selected from public resources, and those labeled "PSM" were developed in our laboratory

^b^^,c^The letters a, c represent early cropping seasons in 2015 and 2016, and b, d represent late cropping seasons in 2015 and 2016, respectively. Detected seasons mean the time when putative QTLs were detected on the SSSLs

Most of these QTLs can be simultaneously detected at the early and late growing seasons, and *qLTG1* (S2), *qLTG3a* (S6), *qLTG5* (S12), *qLTG7b* (S17), *qLTG8b* (S20), *qLTG11b* (S24) were repeatedly detected in three seasons, indicating that these QTLs were stable in different environments. Nevertheless, *qLTG6* (S14) and *qLTG9* (S21) were only identified in the early cropping seasons, while *qLTG2* (S3), *qLTG6* (S15), and *qLTG11a* (S22) were specifically detected in the late cropping seasons (Table 1).

### Substitution Mapping of LTG QTLs

**Fig. 2:**
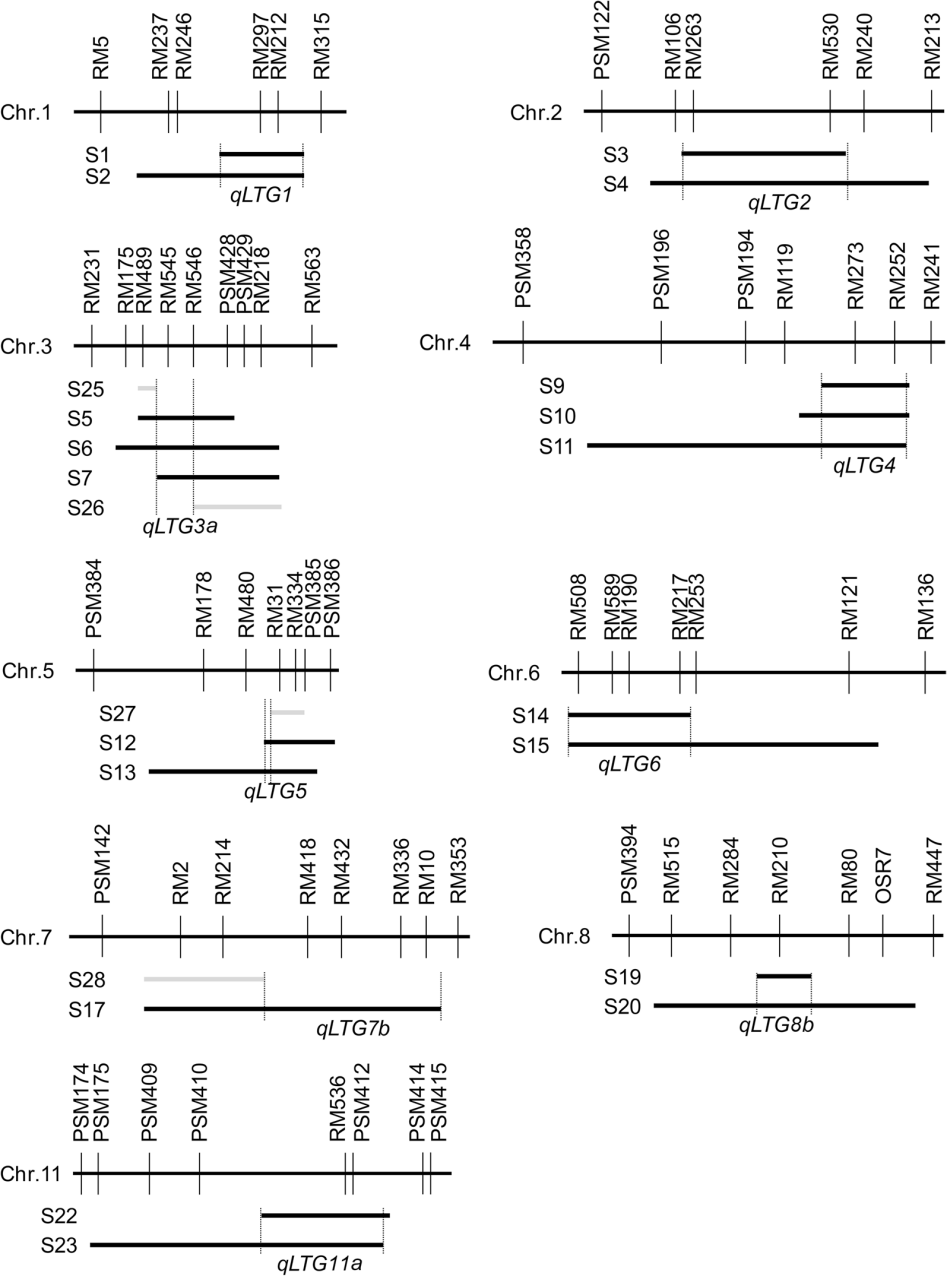
Substitution mapping of LTG QTLs in rice. The long horizontal dark bars represent the chromosomes with the molecular markers above them. The short black horizontal bars coded from S1 to S24 represent the SSSLs detected with QTLs in at least two cropping seasons, while the gray ones coded from S25 to S28 represent the SSSLs not detected with a QTL in all cropping seasons. The intervals located LTG QTLs are shown between two vertical dotted lines with the names of QTLs under the substituted segments

Five QTLs, *qLTG3b*, *qLTG7a*, *qLTG8a*, *qLTG9,* and *qLTG11b,* were detected on a single SSSL. They were located on their corresponding substituted segments with an interval of 6.5–17.1 cM (Table 2). The *qLTG7b* was detected on a single SSSL (S17), but it was not detected on S28 which overlapped with S17, implying that *qLTG7b* was located in the non-overlapping interval of S17 and S28 (Fig. 2; Table 2). However, the remaining eight QTLs were detected simultaneously on multiple SSSLs with overlapping fragments. Therefore, we conducted substitution mapping to integrate these QTLs and reduce their location intervals to smaller genetic regions (Fig. 2; Table 2).

**Table 2:** Integrated LTG QTLs after substitution mapping

QTL	Donor code	Chr	Interval (cM)	Length (cM)	a*	*R*^*2*^ (%)*
*qLTG1*	W14	1	123.1–139.8	16.7	− 0.17 ± 0.01	29.0 ± 0.6
*qLTG2*	W08, W09	2	102.7–132.9	30.2	0.11 ± 0.01	19.5 ± 0.8
*qLTG3a*	W06	3	22.7–29.3	6.6	0.17 ± 0.01	33.0 ± 3.2
*qLTG3b*	W17	3	108.6–125.0	16.4	− 0.10 ± 0.01	19.2 ± 5.8
*qLTG4*	W06	4	67.7–80.6	12.9	− 0.16 ± 0.03	28.5 ± 3.8
*qLTG5*	W01, W11	5	113.9–116.5	2.6	0.14 ± 0.01	28.4 ± 0.5
*qLTG6*	W23, W27	6	0.7–17.3	16.6	0.10 ± 0.07	19.5 ± 15.0
*qLTG7a*	W06	7	18.5–28.5	10.0	− 0.22 ± 0.09	39.4 ± 3.7
*qLTG7b*	W05	7	55.8–80.4	24.6	− 0.24 ± 0.08	39.9 ± 8.9
*qLTG8a*	W06	8	36.5–43.0	6.5	0.15 ± 0.02	26.9 ± 4.4
*qLTG8b*	W06	8	82.6–91.7	9.1	0.16 ± 0.01	31.0 ± 3.7
*qLTG9*	W27	9	48.0–65.1	17.1	− 0.21 ± 0.01	34.2 ± 6.0
*qLTG11a*	W06	11	39.8–59.3	19.5	0.15 ± 0.01	31.3 ± 1.3
*qLTG11b*	W17	11	81.9–97.3	15.4	− 0.19 ± 0.07	34.1 ± 9.7

*a = average additive effect of the QTL from different SSSLs; *R*^*2*^ (%) = average additive effect contribution (%) of the QTL from different SSSLs

*qLTG1* was detected simultaneously on S1 and S2; thus, it was located in the overlapping region in the vicinity of RM297-RM212 with an interval length of 16.7 cM. S3 and S4 carried *qLTG2*, which was delimited to a 30.2 cM overlapping region flanked by RM263 and RM530. *qLTG3a* was detected simultaneously on S5, S6, and S7, but not on S25 and S26, which shared overlapping fragments with the three former SSSLs. *qLTG3a* was hence located in the vicinity of RM545 with an interval length of 6.6 cM. *qLTG4* was detected simultaneously on S9, S10, and S11. Thus, it was mapped in the overlapping region in the vicinity of RM273 with an interval length of 12.9 cM. *qLTG5* was detected simultaneously on S12 and S13, but not on S27, which shared an overlapping region with the other two SSSLs, indicating that *qLTG5* was located in the vicinity of RM480 and RM31 with an interval length of 2.6 cM. *qLTG6* was detected simultaneously on S14 and S15, and thus it was located in the overlapping region RM508-RM217 with an interval length of 16.6 cM. *qLTG8b* was detected simultaneously on S19 and S20, and it was located in the overlapping region in the vicinity of RM210 with an interval length of 9.1 cM. S22 and S23 were demonstrated to carry *qLTG11a* on their overlapping segments in the area of PSM410-PSM412 with an interval length of 19.5 cM.

**Fig. 3:**
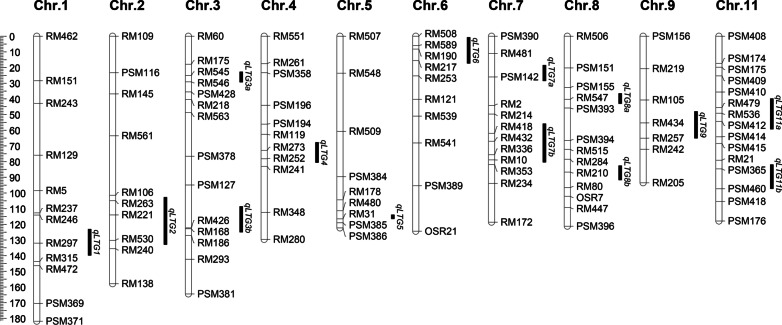
Chromosome location of 14 QTLs for LTG in rice. The linkage map of QTLs was constructed by using MapChart 2.2 (Voorrips 2002). SSR markers are indicated on the right of the chromosomes. Genetic distance (cM) is shown as rulers on the left margin. Black bars on each chromosome's right are the location intervals of QTLs for LTG with their names on the right. *Chr* chromosome

By substitution mapping, 14 integrated LTG QTLs were mapped on ten chromosomes except on chromosomes 10 and 12 (Table 2; Fig. 3). Their locations were narrowed down to shorter intervals ranging from 2.6 to 30.2 cM. The additive effects changed from -0.24 to 0.17, and the additive effect contributions varied from 19.2 to 39.9%. *qLTG2*, *qLTG3a*, *qLTG5*, *qLTG6*, *qLTG8a*, *qLTG8b,* and *qLTG11a* were positive QTLs with excellent alleles for LTG, while the other seven QTLs were detected with negative effects. It is worth noting that the additive effect contributions of *qLTG3a*, *qLTG8b*, and *qLTG11a* are higher than 30%, so they are the main LTG QTLs with utilization values (Table 2).

### Validation and Genetic effect of a Major QTL *qLTG3a*

**Fig. 4:**
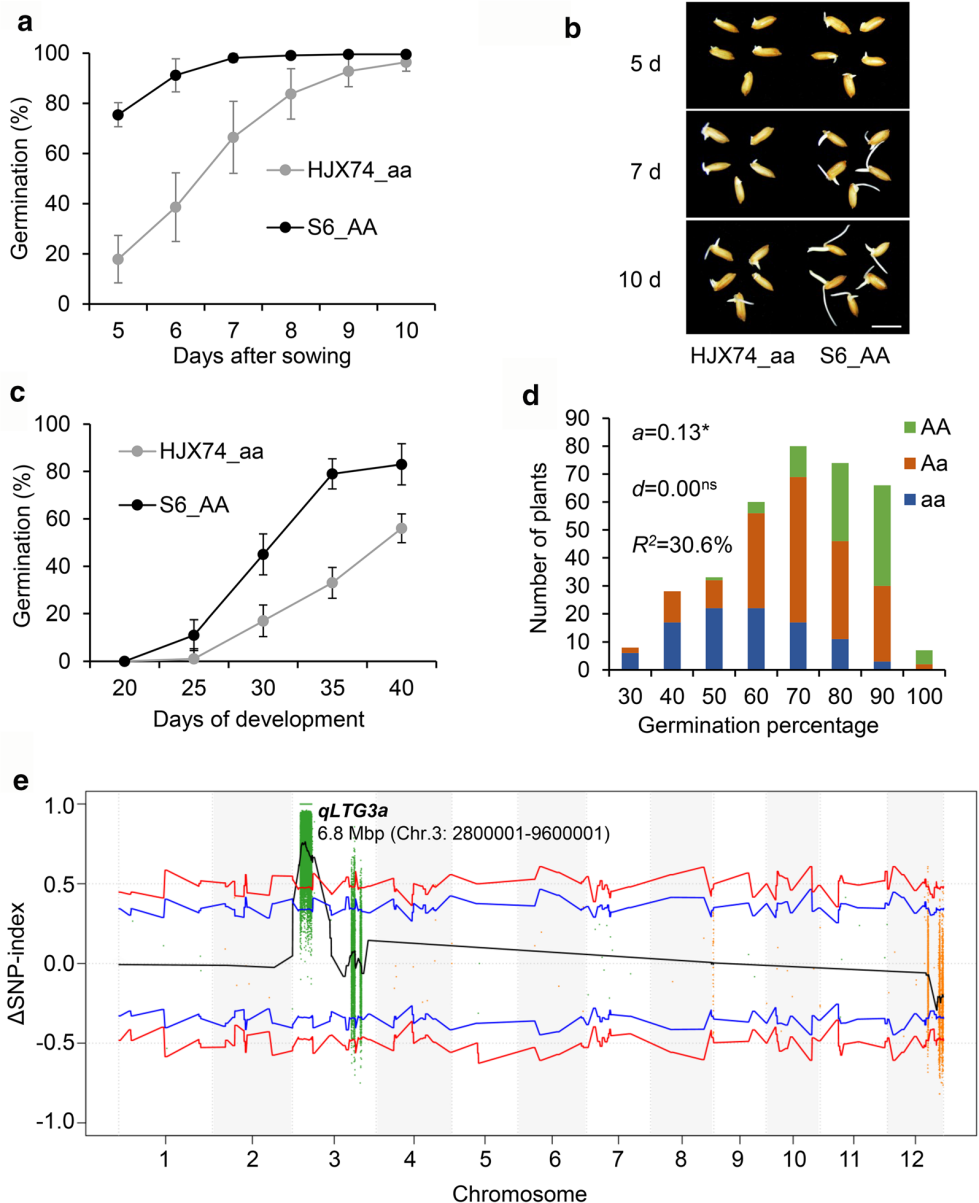
Validate a major LTG QTL, *qLTG3a*, by an F_2_ population and BSA sequencing. "aa" represents the HJX74 allele of *qLTG3a,* and "AA" represents the S6 allele of *qLTG3a*. Roles of *qLTG3a* in LTG during seed germination (**a**) and seed development (**c**). Dots and bars represent the means and standard deviations of LTG at each time point. Germination data were calculated based on at least four biological replicates during seed germination and nine biological replicates during seed development. **b** Representative germination images showing different LTG phenotypes between HJX74 and S6. Pictures were taken on the 5th, 7th, and 10th day after sowing. Bar = 1 cm. **d** Frequency distribution and genetic effects of *qLTG3a* in a 356-progeny population derived from the cross of HJX74 and S6. The donor parent of S6 is Katy. LTG was evaluated on the 7th day after sowing. The genotypes of individual plants were determined by the InDel P7 marker. The additive effects (*a*), dominance effects (*d*), and proportion of the variance explained by the QTL (*R*^2^) were estimated based on Model 1. A positive *a* or *d* value indicates that the "A" allele increased LTG. * for *P* < 0.0001 and ns for *P* ≥ 0.05. **e** Manhattan plot shows the distribution of SNP-index and ΔSNP-index on 12 chromosomes. The reference genome for BSA sequencing is the indica variety R498 (Shuhui498). The blue and red lines represent 95 and 99% confidence intervals, respectively. The black line shows the ΔSNP-index value of fitting results

It indicated that *qLTG3a* was a stable and major LTG QTL, and S6 was selected as the representative SSSL of *qLTG3a* for further study. During seed germination, S6 with the tolerant allele "A" exhibited significantly better low-temperature germination performance than the recipient parent HJX74 carrying the sensitive allele "a", including higher germination rate, faster germination speed, and stronger radicles and plumules (Fig. 4a, b). Moreover, the role of *qLTG3a* on seed development was investigated at the seed maturation stage. As expected, the tolerant allele "A" could remarkably promote the low-temperature germination ability of seeds developing for 25–40 days compared with the sensitive allele "a" (Fig. 4c).

To evaluate the genetic effect of *qLTG3a*, a 356-plants F_2_ population segregating at the QTL was developed from a cross between S6 and HJX74. Normal distribution was observed in the segregating population (Fig. 4d). The average LTG values of plants with genotypes AA, Aa, and aa were 78, 65, and 52%, respectively, and were significantly different. The numbers of plants with genotypes AA, Aa, and aa were 85, 173, 98, respectively, which accorded with the segregation ratio of 1:2:1 (*χ*^2^ = 1.23 < *χ*^2^_0.05,2_ = 5.99) (Fig. 4d). A significant positive additive effect of *qLTG3a* was detected in the segregating population with a phenotypic contribution of 30.6%. However, the dominance effect of *qLTG3a* was not identified, suggesting it is a purely additive allele for LTG (Fig. 4d). Moreover, *qLTG3a* was further confirmed by BSA-seq using the above F_2_ segregation population (Fig. 4e). At the 95% significance level, only one QTL interval was identified on chromosome 3 by the ΔSNP-index method after the whole genome screening. The corresponding position on the R498 genome was Chr.3: 2800001–9600001 bp, a 6.8-Mb region, which is completely located on the substitution segment of S6 (Fig. 4e). Therefore, this QTL detected by BSA-seq was considered as the same one of *qLTG3a* identified by the conventional method.

### Fine Mapping of *qLTG3a*

**Fig. 5:**
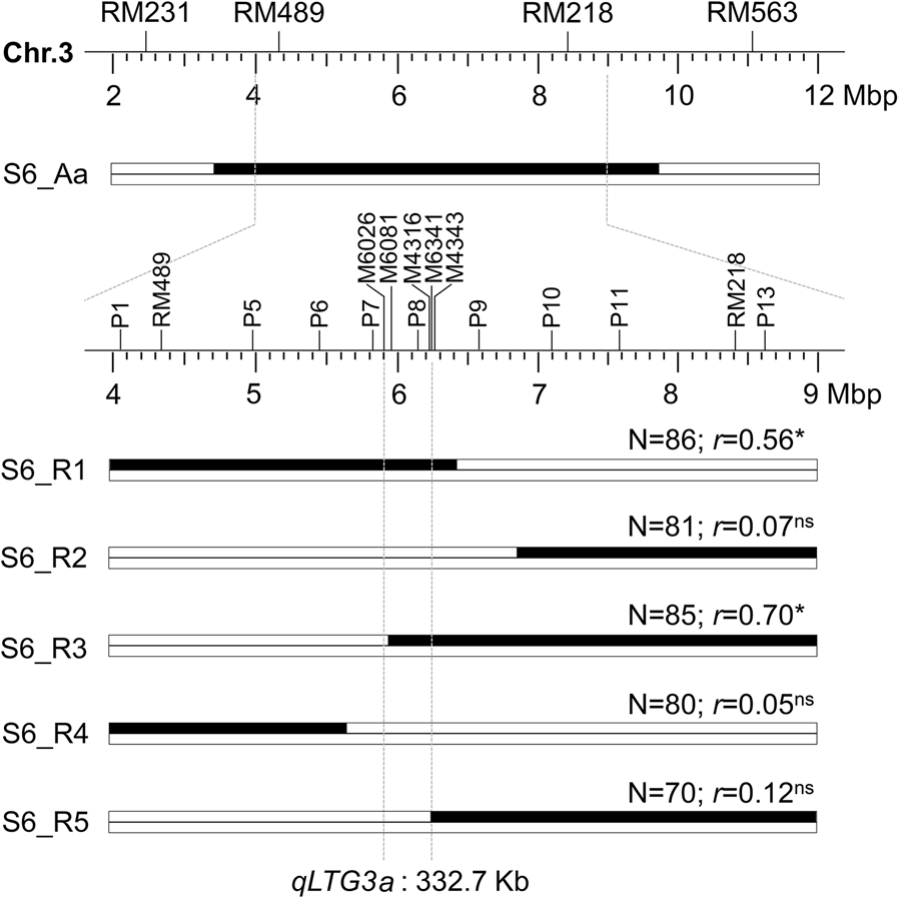
Fine mapping of *qLTG3a*. Five recombinants (S6_R1 to R5) derived from S6_Aa were selected to delimit the locus of *qLTG3a* to a 332.7-kb region flanked by M6026 and M6341. The dotted vertical lines indicate the target region of *qLTG3a*. The partial physical maps of chromosome 3 were constructed based on the Nipponbare reference genome (IRGSP-1.0). Black and white bars represent the chromosomal segments from S6 and HJX74, respectively. N, the number of plants in a recombinant-derived progeny population; *r*, marker-trait correlation coefficients for LTG. A significant *r* value (* for *P* < 0.01 and ns for *P* ≥ 0.05) indicates that the allele of S6 on the marked heterozygous region enhanced LTG

Five recombinants selected from the F_2_ population with crossovers between the flanking markers of *qLTG3a* were advanced to the F_3_ generation to conduct a progeny test (Fig. 5). The partial physical map was constructed with 14 new InDel markers, which were applied to determine the recombinants' points of crossovers. Progeny populations (70–86) were genotyped by P1, P8, or P13 segregating in the corresponding populations and phenotyped by testing LTG.

Significant associations between marker genotypes and trait values of LTG were only detected in S6_R1 and S6_R3 progeny populations, with *r* = 0.56 to 0.70 (Fig. 5). Meanwhile, genetic analysis confirmed that the narrowed *qLTG3a*-containing interval had significant additive effects in S6_R1 and S6_R3 progeny populations, and also a minor dominance effect in the S6_R3 progeny population, which together accounted for 33% to 53% of the phenotypic variances (Additional file 1: Table S5). Besides, the absence of marker-trait association and the significant additive effect of *qLTG3a* was observed in the progeny lines of S6_R2, S6_R4, and S6_R5 (Fig. 5; Additional file 1: Table S5). According to the substitution mapping method, *qLTG3a* was narrowed down to a 332.7-kb heterozygous region of S6_R1 and S6_R3 flanked by M6026 and M6341.

### Candidate Genes Underlying *qLTG3a*

**Fig. 6:**
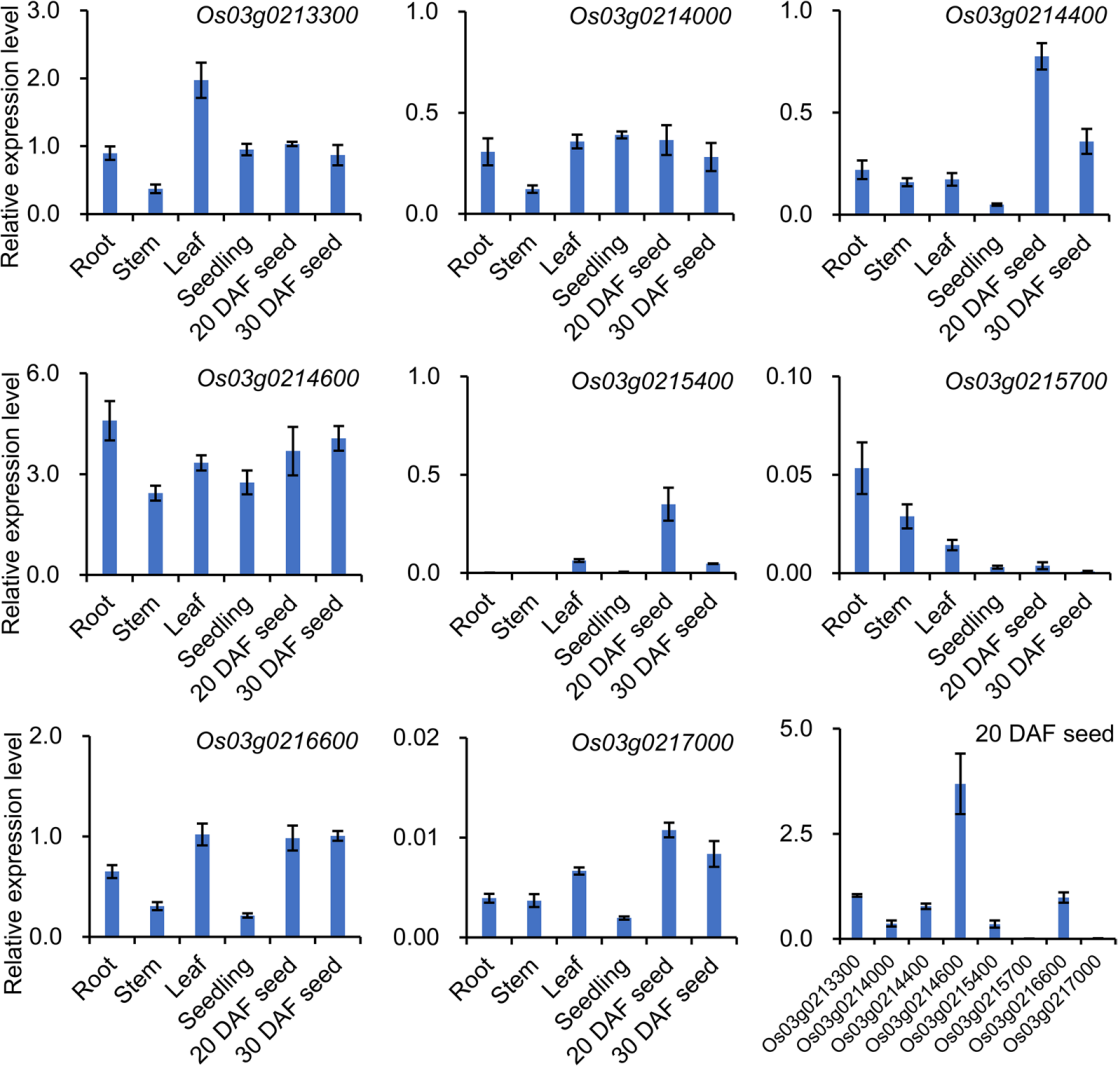
Expression patterns of the candidate genes underlying *qLTG3a* in various tissues. Root, stem, and leaf were collected at the reproductive stage, and seedlings were three days old. The root sample of *Os03g0213300* was set as control sample for all genes. Transcript levels were assessed by qRT-PCR using *OsActin1* as an internal control. Data are means ± SD (n = three biological replicates). DAF, days after flowering

Within the 332.7 kb region of *qLTG3a*, 40 annotated genes were identified based on the RAP-DB database (https://rapdb.dna.affrc.go.jp) (Table 3). Among these genes, 11 genes were predicted as hypothetical proteins or genes which were excluded from the list of candidate genes, while the other 29 genes showed functional annotations. In addition, BSA-seq data were used to reveal the sequence variations, and only the nonsynonymous SNPs and InDels (ΔSNP/InDel-index > 0.8) in the promoter and coding regions were selected for further analysis. Under this standard, among the 29 genes, a total of 33 sequence variations were detected between the two parents of S6 and HJX74, corresponding to 16 candidate genes (Table 3, Additional file 1: S7, S8). To further reduce the number of candidate genes, we analyzed the temporal and spatial expression patterns of the above 16 genes. Gene expression analysis by RiceXPro indicated that four genes, *Os03g0213300*, *Os03g0214000*, *Os03g0214400*, and *Os03g0214600*, were highly expressed in seeds (Table 3; Additional file 1: Fig. S3). Moreover, similar expression patterns were obtained after we investigated the expression of eight genes by qRT-PCR (Fig. 6). In mature developing seeds (20-30 DAF), *Os03g0213300*, *Os03g0214400*, *Os03g0214600*, and *Os03g0216600* showed high expression levels, *Os03g0214000* and *Os03g0215400* displayed medium expression levels, while the expression levels of *Os03g0215700* and *Os03g0217000* were low (Fig. 6). Since *Os03g0216600* has no sequence variation, *Os03g0213300*, *Os03g0214400*, and *Os03g0214600* were considered as the final possible candidate genes responsible for LTG.

**Table 3:** Function annotation, sequence variation and gene expression of candidate genes in the *qLTG3a* region

Gene RAP ID^a^	Gene function ^b^	Indel number*	SNP number*	Gene expression level in seeds ^c^
*Os03g0213300*	Phosphopantetheine attachment site domain containing protein; Hypothetical conserved gene	1	3	High
*Os03g0213400*	Similar to RNA helicase			Medium
*Os03g0213500*	Conserved hypothetical protein			High
*Os03g0213600*	Conserved hypothetical protein	3	3	Medium
*Os03g0213700*	DUF647 domain containing protein	1		Medium
*Os03g0213800*	Mitochondrial substrate carrier family protein	2		Medium
*Os03g0213900*	Conserved hypothetical protein; ROUGH SHEATH2-interacting KH-domain protein	3	3	Medium
*Os03g0214000*	Diphosphonucleotide phosphatase 1 precursor; nucleotide pyrophosphatase	1		High
*Os03g0214050*	Hypothetical protein			no data
*Os03g0214100*	Replication protein A1		1	Low
*Os03g0214200*	Mediator of OsbZIP46 deactivation and degradation, Negative regulation of ABA signaling and drought tolerance; Ninja-family protein 1			Medium
*Os03g0214400*	Digalactosyldiacylglycerol synthase 2	4		High
*Os03g0214600*	26S proteasome subunit RPN9a; 26S proteasome non-ATPase regulatory subunit 13	2		High
*Os03g0214900*	Conserved hypothetical protein	4	2	Medium
*Os03g0215000*	Integral membrane family protein			High
*Os03g0215200*	Putative transcription factor, Carpel specification, Midrib formation			Low
*Os03g0215400*	MADS-domain-containing protein, sexual reproduction	1		Medium
*Os03g0215600*	Zinc finger, CCHC retroviral-type domain containing protein			Very low
*Os03g0215700*	Myosin II heavy chain-like family protein	3	1	Very low
*Os03g0215800*	Pyridoxal phosphate-dependent enzyme	1		Low
*Os03g0215900*	67kD chloroplastic RNA-binding protein, P67			Low
*Os03g0216000*	Zinc-finger protein KNUCKLES			Very low
*Os03g0216300*	Pentatricopeptide repeat domain containing protein; Hypothetical conserved gene	1		Low
*Os03g0216400*	Pentatricopeptide repeat domain containing protein			Low
*Os03g0216500*	Conserved hypothetical protein		1	Low
*Os03g0216600*	Alpha-glucosidase (EC 3.2.1.20)			High
*Os03g0216700*	Citrate transporter, Efficient translocation of Fe	1		Medium
*Os03g0216733*	Hypothetical protein			no data
*Os03g0216766*	Hypothetical gene			no data
*Os03g0216800*	Polygalacturonase B (Fragment)	1	1	Very low
*Os03g0216900*	Prefoldin domain containing protein			Low
*Os03g0217000*	Inhibin beta B chain precursor		1	Low
*Os03g0217200*	Cyclin-like F-box domain containing protein			Medium
*Os03g0217400*	Conserved hypothetical protein			Low
*Os03g0217801*	Hypothetical gene			no data
*Os03g0217900*	Hypothetical protein			Medium
*Os03g0218100*	Sec63 domain containing protein			no data
*Os03g0218200*	F-box domain containing protein	1		Low
*Os03g0218300*	Conserved hypothetical protein		3	Medium
*Os03g0218400*	Similar to Hexose transporter			Low

*Sequence variation analysis of candidate genes was derived from BSA sequencing data. The number of InDels was derived from the upstream and exonic regions of the candidate genes, and the number of SNPs was derived from the nonsynonymous SNPs in the exonic regions. The ΔSNP-index and ΔInDel-index were greater than 0.8

^a^^,b^RAP ID and gene function are based on the Rice Annotation Project Database (RAP-DB): http://rapdb.dna.affrc.go.jp

^c^The expression level of candidate genes in seeds was analyzed by the public tool RiceXPro (http://ricexpro.dna.affrc.go.jp/) and divided into four levels

### *qLTG3a* Enhances Seed Germination Vigor Under Optimal Temperature and Osmotic Stress

**Fig. 7:**
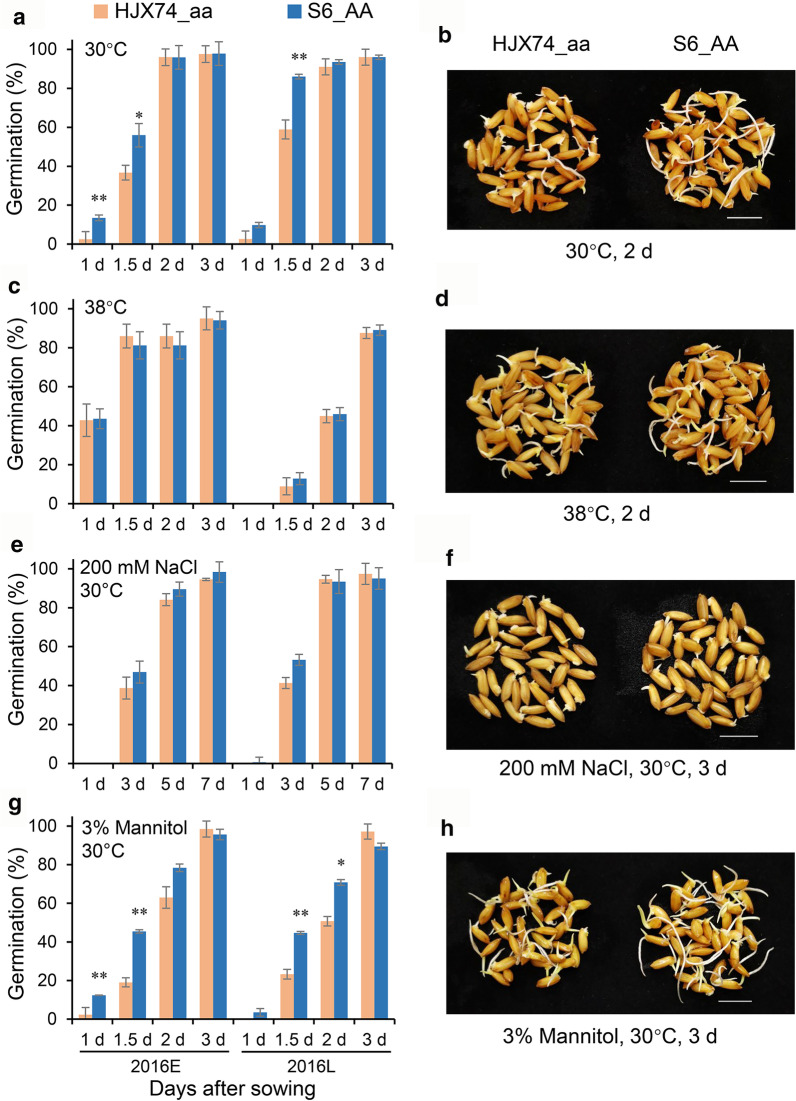
Roles of *qLTG3a* in various environmental stress tolerance. **a**,** b** normal temperature, **c**,** d** high temperature, **e**,** f** high-salt stress, **g**,** h** osmotic stress. "aa" represents the sensitive allele of *qLTG3a* from HJX74 and "AA" represents the tolerant allele of *qLTG3a* from S6. At each time point, columns and bars represent the means and standard deviations of germination rates calculated based on three biological replicates. The significance of differences was calculated using Student's *t* test. *P < 0.05 and **P < 0.01. 2016E, early cropping season in 2016; 2016L, late cropping season in 2016. Bars = 1 cm

To comprehensively reveal the role of *qLTG3a*, we analyzed the germination performance of S6 with tolerant allele "A" and HJX74 with sensitive allele "a" responding to different environmental conditions in two cropping seasons (Fig. 7). In addition to low-temperature stress, the tolerant allele "A" also promoted seed germination ability under optimal temperature (30℃) and osmotic pressure (3% Mannitol) compared with the sensitive allele "a". At 30℃, the germination rates of S6_AA were significantly higher than that of HJX74_aa at 1 to 1.5 d in 2016E and 1.5 d in 2016L (Fig. 7a, b). Similar germination behavior was also observed after treatment with 3% Mannitol. For example, the germination rates of S6_AA at 1.5 d in 2016E and 2016L were 45.3% and 44.6%, respectively. By contrast, the corresponding germination rates of HJX74_aa were only 19.0% and 23.3%, respectively (Fig. 7g, h). However, S6_AA and HJX74_aa showed similar germination patterns under 38℃ high temperature and 200 mM-NaCl stress, indicating that *qLTG3a* did not work under these two adversities (Fig. 7c–f).

## Discussion

Low temperatures limit the geographical distribution and planting time of crops, thus affecting food production. Reduced freshwater resources and increasing labor costs have driven the rise of direct-seeded rice in China and worldwide (Lv et al. 2020; Mahender et al. 2015). Genetic improvement of LTG for current rice varieties is a crucial strategy to ensure direct-seeded rice success. As previously reported, LTG is a typical quantitative trait influenced by genetic factors and environmental cues, such as temperature and heading stage (Fujino et al. 2008; Jiang et al. 2017; Li et al. 2013; Wang et al. 2018b, c; Xie et al. 2014). An important prerequisite for QTL mapping is to control environmental factors to improve the accuracy of phenotypic identification. Due to the similar genetic background, the SSSL population showed uniform heading dates, and thereby the seeds can be harvested on the same days after heading to keep the consistent maturity state. Meanwhile, only those SSSLs that can repeatedly detect QTLs in two or more seasons will be selected for subsequent QTL analysis to reduce environmental impact (Table 1).

In this study, 208 multi-donor-derived rice SSSLs were used to conduct an integrated QTL analysis of LTG by substitution mapping over four planting seasons. As expected, a total of 14 LTG QTLs were identified (Table 2; Fig. 3), further demonstrating that SSSL is a powerful tool for the analysis of complex agronomic traits (Wang et al. 2012; Wu et al. 2020; Zhou et al. 2017; Zhu et al. 2018). By comparing chromosome positions and molecular markers, 11 LTG QTLs mapped in this study were similar to those of previous QTLs, which proves the reliability of this experimental system. Most of the QTLs are stably identified in both early and late cropping seasons, especially *qLTG1* (S2), *qLTG3a* (S6), *qLTG5* (S12), *qLTG7b* (S17), *qLTG8b* (S20), *qLTG11b* (S24) that were detected in three cropping seasons (Table 1). Interestingly, some QTLs were identified only in early or late cropping seasons, such as *qLTG6* (S14), *qLTG9* (S21), and *qLTG2* (S3), *qLTG6* (S15), *qLTG11a* (S22) separately (Table 1). The season-specific QTLs may correlate with environment temperature and can be applied in specific ecological areas. It is worth noting that *qLTG6* derived from S14 or S15 was specifically detected in the early or late planting seasons, respectively, showing distinct expression patterns. This implies that multiple alleles from different donor sources at the *qLTG6* locus have an apparent seasonal preference in expression. It is generally believed that *japonica* rice, mainly distributed in temperate regions, is more tolerant to low temperatures than *indica* rice widely distributed in tropical and subtropical areas due to habitat adaptability (Jiang et al. 2017; Yang et al. 2019). Consistently, a total of 14 SSSLs with positive-effect QTLs were identified, of which eight SSSLs were derived from a *japonica* cultivar Katy (W06), corresponding to four QTLs, *qLTG3a*, *qLTG8a*, *qLTG8b*, *qLTG11a*, respectively (Table 1, Additional file 1: S1). This result indicates that Katy has abundant cold tolerance genes, strengthening our understanding that *japonica* varieties have excellent cold tolerance.

Four QTLs, *qLTG7b*, *qLTG8b*, *qLTG9* and *qLTG11b*, displayed large additive effect contributions of 39.9%, 31.0%, 34.2% and 34.1%, respectively (Table 2). However, only *qLTG8b* had a positive additive effect, and it corresponds well to *qLTG-8-1* (Jiang et al. 2005), which were linked to the same marker RM210. Li et al. (2013) fine-mapped *qLTG-9* to a 72.3-kb region between makers L9-25d and ID-1, which was located on the segment of RM105-RM434 of *qLTG9* identified in this study (Fig. 3). Noticeably, three novel QTLs, *qLTG3a*, *qLTG7a*, and *qLTG11a*, were identified for the first time in this study. *qLTG3a* and *qLTG11a* displayed positive additive effects and large additive effect contributions of 33% and 31%, respectively (Table 2). Also, *qLTG3a* can be detected repeatedly in different seasons and multiple SSSLs (Table 1). According to the distinctly different chromosomal locations, it is evident that there is no correlation between *qLTG3a* identified in this study and the first map-based cloned gene *qLTG3-1* reported by Fujino et al. (2008). Because the former is located at approximately 25 cM of chromosome 3, closely linked to RM545 (Fig. 3), while the latter is located near the end of chromosome 3 in the vicinity of GBR3001 (Fujino et al. 2004). Therefore, *qLTG3a* represents a new, main-effect, stably expressed LTG QTL, which can be used as a new candidate gene for breeders to cultivate elite varieties with LTG traits.

To verify *qLTG3a*, we further examined whether it could enhance LTG during seed germination and development. As expected, S6 carrying the "AA" tolerance gene showed significantly improved seed vigor under low-temperature conditions (Fig. 4a–c). Subsequently, we estimated the genetic effects of *qLTG3a* through an F_2_ segregating population and confirmed the location of *qLTG3a* by BSA-seq (Fig. 4d, e). Finally, *qLTG3a* was fine mapped to a 332.7-kb physical region by the progeny derived from five recombinants (Fig. 5). Forty candidate genes were revealed based on function annotation, and three most likely candidate genes were identified by gene sequence and expression analysis (Table 3; Fig. 6). *Os03g0213300* encodes a phosphopantetheine attachment site domain containing protein, *Os03g0214400* encodes a digalactosyldiacylglycerol synthase 2, and *Os03g0214600* encodes a 26S proteasome subunit RPN9a. It lays a foundation for dissecting the molecular basis underlying *qLTG3a*. Currently, only two natural variations related to LTG, *qLTG3-1* (Fujino et al. 2008) and *OsSAP16* (Wang et al. 2018c), have been cloned by map-based strategy and GWAS, respectively. *qLTG3-1* may reduce the mechanical resistance during seed germination by inducing programmed cell death in the covered tissues of the embryo (Fujino et al. 2008).

In addition to low temperature, *qLTG3-1* also significantly promoted seed germination at the optimal temperature and responded to various stresses such as high salt, high osmolarity, and high temperature (Fujino et al. 2008). Similarly, we also found that *qLTG3a* identified in this study could also play a role under normal temperature and high osmotic stress, but did not respond to high temperature and high salt stresses (Fig. 7). Therefore, it is reasonable to speculate that plant cells have established some conservative mechanisms in response to low temperature and osmotic stress. Previous studies have shown that rice accumulates a large number of soluble sugars, including glucose, fructose, sucrose, hexose, raffinose, and trehalose under low-temperature stress, thereby improving the ability of cells to cope with osmotic stress (Liu et al. 2018). In the future, we will explore the possible relationship between *qLTG3a* and soluble sugars to enrich our understanding of the molecular mechanism of LTG.

In summary, we identified 14 QTLs for LTG by substitution mapping from 208 SSSLs in four seasons, fine mapped *qLTG3a* and identified three key candidate genes. All these results will help us dissect the genetic mechanisms of LTG and provide elite candidate genes to cultivate new high-vigor rice varieties with low-temperature germination resistance.

## Supplementary Information

**Additional file 1**: **Figure S1.** Length (a) and genome coverage (b) of substituted segments in 208 rice single segment substitution lines (SSSLs). cM, centimorgan. **Figure S2.** Phenotypic variation of LTG between recipient parent HJX74 and different SSSLs. S6, S15, S22 were three representative SSSLs with good LTG values. Pictures were taken on the 6th, 8th, and 10th day after sowing. Bars=1 cm. **Figure S3**. Temporal and spatial expression profiles of candidate genes in *qLTG3a* region. All expression data were prepared by the public tool RiceXPro (http://ricexpro.dna.affrc.go.jp/). The grey boxes mean no expression data. The heat map was made by TBtools (v1.089). **Table S1.** Summary the donors and substituted segments distribution of 208 SSSLs. **Table S2.** The sequence of primers used in this study. **Table S3.** ANOVA analysis of the mean LTG values of HJX74 in different blocks of each cropping season. **Table S4.** LTG of HJX74 and 24 SSSLs at 15°C in four cropping seasons. **Table S5.** Summary of genotypic means for germination and genetic effects of markers segregating in progeny lines derived from selected recombinants for *qLTG3a* region. **Table S6.** The RAP ID, MSU ID and R498 (Shuhui498) ID of candidate genes. **Table S7.** Nonsynonymous SNP variations (ΔSNP-index > 0.8) of candidate genes in the *qLTG3a* locus. **Table S8.** Indel variations (ΔInDel-index > 0.8) in the upstream and exonic regions of candidate genes in the *qLTG3a* locus.

## Data Availability

The datasets supporting the conclusions of this article are included within the article and its additional files.

## Abbreviations

BSA: Bulk segregant analysis
GWAS: Genome-wide association study
HJX74: Huajingxian 74
InDels: Insertion-deletions
LTG: Low-temperature germinability
SNP: Single-nucleotide polymorphisms
SSSL: Single segment substitution line
