# Supplementary material for: Integrative QTL Identification, Fine Mapping and Candidate Gene Analysis of a Major Locus qLTG3a for Seed Low-Temperature Germinability in Rice
Source: Rice (N Y). 2021 Dec 15;14:103. doi: 10.1186/s12284-021-00544-2 (PMC8674402; doi:10.1186/s12284-021-00544-2)
Supplement: Supplementary file 1 — Additional file 1: Figure S1. Length (a) and genome coverage (b) of substituted segments in 208 rice single segment substitution lines (SSSLs). cM, centimorgan. Figure S2. Phenotypic variation of LTG between recipient parent HJX74 and different SSSLs. S6, S15, S22 were three representative SSSLs with good LTG values. Pictures were taken on the 6th, 8th, and 10th day after sowing. Bars=1 cm. Figure S3. Temporal and spatial expression profiles of candidate genes in qLTG3a region. All expression data were prepared by the public tool RiceXPro (http://ricexpro.dna.affrc.go.jp/). The grey boxes mean no expression data. The heat map was made by TBtools (v1.089). Table S1. Summary the donors and substituted segments distribution of 208 SSSLs. Table S2. The sequence of primers used in this study. Table S3. ANOVA analysis of the mean LTG values of HJX74 in different blocks of each cropping season. Table S4. LTG of HJX74 and 24 SSSLs at 15°C in four cropping seasons. Table S5. Summary of genotypic means for germination and genetic effects of markers segregating in progeny lines derived from selected recombinants for qLTG3a region. Table S6. The RAP ID, MSU ID and R498 (Shuhui498) ID of candidate genes. Table S7. Nonsynonymous SNP variations (ΔSNP-index > 0.8) of candidate genes in the qLTG3a locus. Table S8. Indel variations (ΔInDel-index > 0.8) in the upstream and exonic regions of candidate genes in the qLTG3a locus. [file 12284_2021_544_MOESM1_ESM.pdf]

Integrative QTL Identification, Fine Mapping and Candidate Gene Analysis of a Major Locus *qLTG3a* for Seed Low-temperature Germinability in Rice

Authors:

Zhaoyuan Pan[1†], Bin Tan[1†], Guiyuan Cao[1], Rongqi Zheng[1], Meng Liu[1], Ruizhen Zeng[1], Shaokui Wang[1], Haitao Zhu[1], Heng Ye[2], Guangmiao Zhao[1], Wei Cao[1], Guifu Liu[1], Guiquan Zhang[1*] and Yuliang Zhou[1*]

Institution address:

[1] Guangdong Key Laboratory of Plant Molecular Breeding and State Key Laboratory for Conservation and Utilization of Subtropical Agro-Bioresources, South China Agricultural University, Guangzhou 510642, China

[2] Division of Plant Sciences, University of Missouri, Columbia, MO 65211, USA

† Zhaoyuan Pan and Bin Tan contributed equally to this work.

[*] **Corresponding author 1:**

Name: Guiquan Zhang

E-mail: gqzhang@scau.edu.cn

Tel: +86-20-85281175

Fax: +86-20-85281175

[*] **Corresponding author 2:**

Name: Yuliang Zhou

E-mail: zhouyuliang@scau.edu.cn

Tel: +86-13430221525

Fax: +86-20-85280203

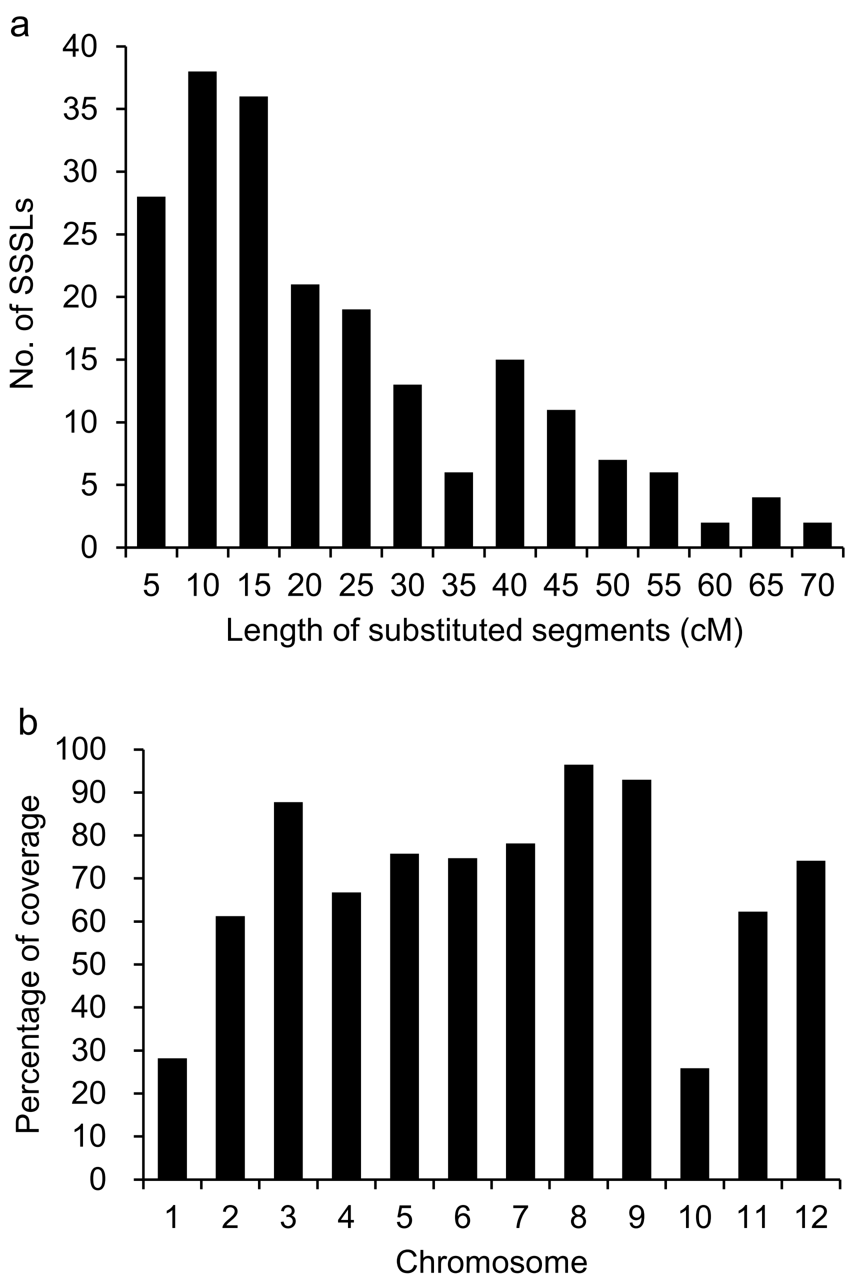

Figure S1 Length (a) and genome coverage (b) of substituted segments in 208 rice single segment substitution lines (SSSLs). cM, centimorgan

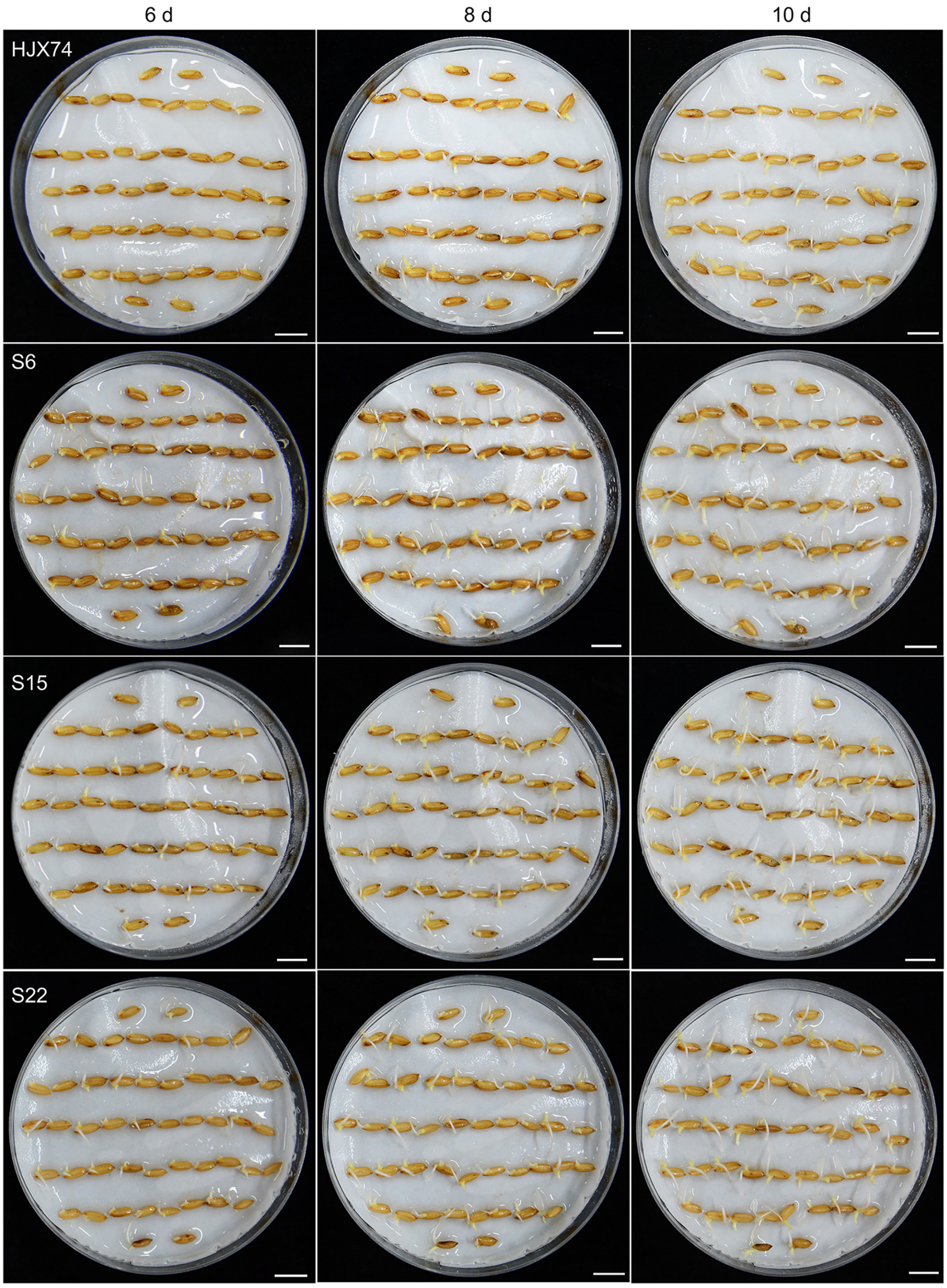

Figure S2 Phenotypic variation of LTG between recipient parent HJX74 and different SSSLs. S6, S15, S22 were three representative SSSLs with good LTG values. Pictures were taken on the 6th, 8th, and 10th day after sowing. Bars=1 cm

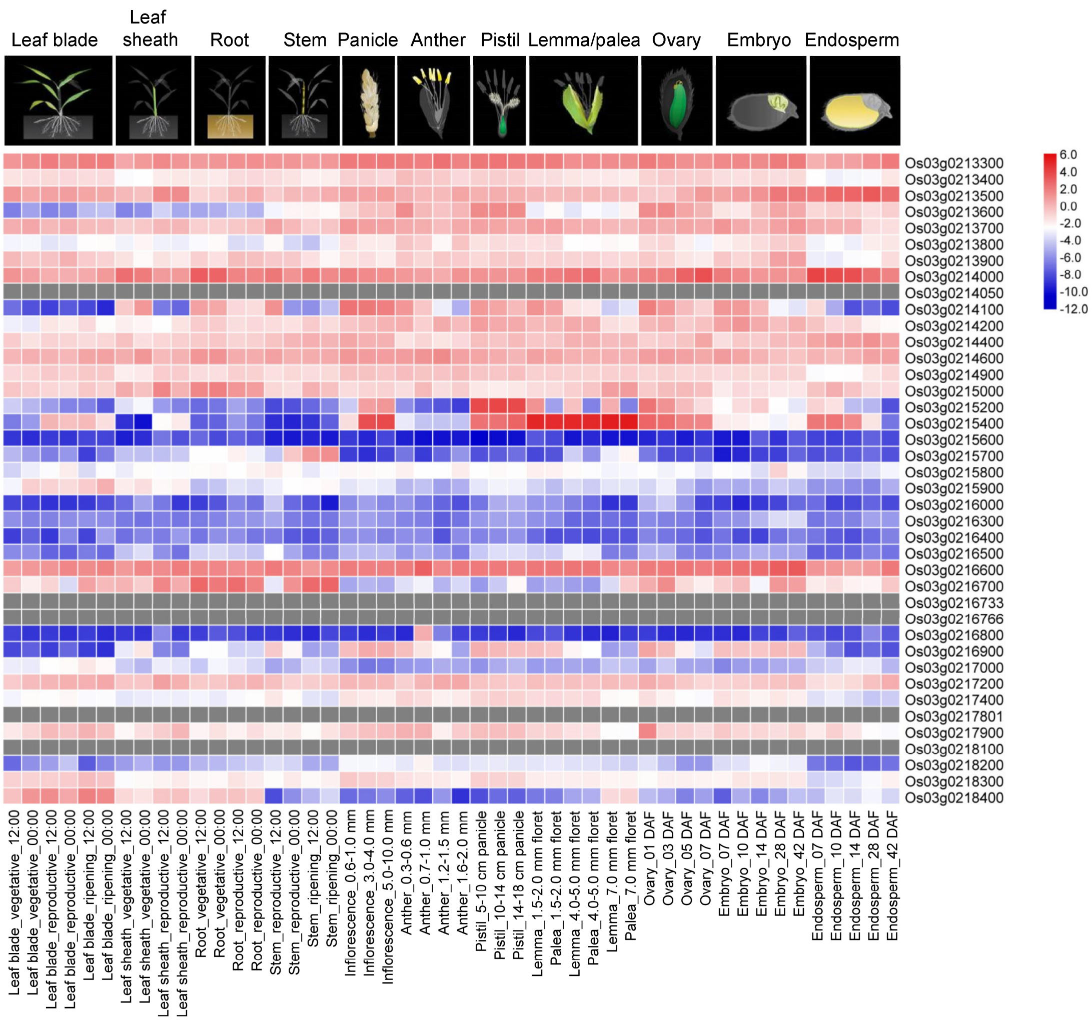

Figure S3 Temporal and spatial expression profiles of candidate genes in *qLTG3a* region. All expression data were prepared by the public tool RiceXPro (http://ricexpro.dna.affrc.go.jp/). The grey boxes mean no expression data. The heat map was made by TBtools (v1.089).

Table S1 Summary the donors and substituted segments distribution of 208 SSSLs

Donor	Code	Type	Chromosome												Total
			1	2	3	4	5	6	7	8	9	10	11	12	
Tetep	W01	I					2	2							4
Amol 3	W02	I		1							1				2
Zhong4188	W03	I						5							5
BG367	W04	I						1	5		3				9
Zihui 100	W05	I		4	1	4	2	4	10	5	2				32
Katy	W06	J	4	1	7	5		1	4	14	7	2	5	2	52
Suoyunuo	W07	J		1			1	2	2						6
IR64	W08	I		3			2	2							7
Basmati 385	W09	I		4											4
Nanyangzhan	W10	J					1								1
Basmati 370	W11	I		3		1	1	2							7
IR58025B	W12	I						3							3
Jiangxisimiao	W13	I						1							1
Lianjian33	W14	I	6	1			1	4				1	1		14
American Jasmine	W15	I				1									1
Ganxiangnuo	W17	I		1	3		1	7	3	1		1	2	9	28
IRAT261	W18	J		1		1	2								4
Chenglong shuijingmi	W20	I		1		1		1							3
IR65598-112-2	W21	J						5							5
Khazar	W22	J		2											2
Lemont	W23	J						2			1				3
Star bonnet 99	W24	J						2							2
IAPAR9	W27	J		3			4	5							12
IR66897B	W31	J						1							1
Total			10	26	11	13	17	50	24	20	14	4	8	11	208

I: *Indica*; J: *Japonica*

Table S2 The sequence of primers used in this study

Marker name	Forward primers	Reverse primers	Position (bp)	Expected size (bp)	Purpose
RM231	CACTTGCATAGTTCTGCATTG	CCAGATTATTTCCTGAGGTC	2454089	191	Fine mapping
P1	GTTCATGTCCATCTCGATCT	GTAGTTCGAGCACCAATCTC	4061303	167	Fine mapping
RM489	TCACCCATGGATGTTGTCAG	ACTTGAGACGATCGGACACC	4334678	271	Fine mapping
P5	GAGTAGTTGTGTTTTGGGGA	ATCGATAGAGCCCACAGATA	4984938	176	Fine mapping
P6	GAACCGATCGAGATACGTC	CCCCTTGACCTGAGATTACT	5443380	179	Fine mapping
P7	GGTTCACTGACCTCCATTAC	GTGCCTTCTCCTTCTCCTAC	5823494	184	Fine mapping
M6026	TGCAATCAACCGCTCGAGTA	GTAGCATCAGCATCAGGGAGT	5903114	289	Fine mapping
M6081	AGTCGCGTGTTGGTAGTAGT	TATAACACTCGCACAGATGC	5959852	151	Fine mapping
P8	GGCTCGAAACAGGTAGGTA	AACCGAGAGAAGCAGCAG	6142649	146	Fine mapping
M4316	GATCCGCTTCACCTTCAC	CCTCCTCCTCTCTGTTTTTC	6227387	161	Fine mapping
M6341	CTCTCCTACCAATCAAGCAC	TTTGATTTTTAAGCAGAGGC	6235761	140	Fine mapping
M4343	CCTGATACGTGGTAGGAATC	TAAAAATGACCAAAATGCCT	6258955	158	Fine mapping
P9	GTAACCAAGGAACCCTTTTT	TAAGGAAGCAACCAAAGAGA	6577282	155	Fine mapping
P10	TTGTCCTACTTCCTGCATTT	TAAATTTTCAGGGACTTTGG	7091304	193	Fine mapping

Table S2 (continued)

Marker name	Forward primers	Reverse primers	Position (bp)	Expected size (bp)	Purpose
P11	TTCCAAAACTTTCTTGTCGT	AGAATAAGAGGCAGCAACAG	7582786	140	Fine mapping
RM218	TGGTCAAACCAAGGTCCTTC	GACATACATTCTACCCCCGG	8406430	149	Fine mapping
P13	CTGACATACAAGCAATGTGG	ATTAGAATCGACAAACACGG	8624242	164	Fine mapping
RM563	CGACCCTAGGGTTTCTCC	CTCGACGTCGTGGAAAGC	11071952	186	Fine mapping
Os03g0213300 qRT	TGTTCCAAATAGCTCCAGTTC	GTTGAATTGTCCACCTTATGC	NA	120	qRT-PCR
Os03g0214000 qRT	CTACTTGTCCATCAGAGAGC	CTGAAGCTTCATGGATCCC	NA	128	qRT-PCR
Os03g0214400 qRT	CTATGTTCGTCGCTGGATTG	AGCCACTTCATCTGGAATAC	NA	142	qRT-PCR
Os03g0214600 qRT	GGAATTGTTAGCTCACCCGA	ACTGAGAGCAGCATTGTGGA	NA	139	qRT-PCR
Os03g0215400 qRT	GAAACCAGTGCAGAGAATGTG	CTGGTGATGGTGAGCATGA	NA	180	qRT-PCR
Os03g0215700 qRT	ATGCCTGTCAAGAGATGGC	GCTAGCAGACGTGTTGGAT	NA	186	qRT-PCR
Os03g0216600 qRT	ACAACTCTGCAGATTGGGAC	TACTTGGTACCAACGCACCA	NA	146	qRT-PCR
Os03g0217000 qRT	AGATCATCGTCCTTCCTCTG	AAGGAGAAGCTCATGAGCG	NA	248	qRT-PCR
OsActin1 qRT	CGGGAAATTGTGAGGGACAT	AGGAAGGCTGGAAGAGGACC	NA	181	qRT-PCR

NA: not applicable

Table S3 ANOVA analysis of the mean LTG values of HJX74 in different blocks of each cropping season

Blocks	Germination (%)			
	2015E	2015L	2016E	2016L
1	66.6±3.8	53.2±1.7	65.8±2.2	38.6±3.1
2	72.5±3.6	51.6±2.4	52.7±0.5	45.2±5.9
3	49.7±4.6	63.8±1.0	53.6±1.7	39.6±1.1
4	68.8±2.8	47.5±4.1	55.3±2.9	39.6±6.9
5	66.6±2.5	62.4±2.8	60.3±2.6	37.1±1.5
6	70.3±4.4	62.2±0.6	64.9±0.7	55.9±3.6
7	79.7±3.8	60.1±5.8	53.4±4.4	46.2±1.8
8	65.9±2.0	65.4±3.7	47.7±4.3	28.9±5.3
9	75.5±0.3	50.2±7.4	43.6±7.0	50.8±3.1
10	61.4±7.0			55.1±5.9
11				47.9±4.4
P value	1.52E-01	4.57E-01	3.59E-01	4.98E-01

2015E, 2015L, 2016E and 2016L represent the early (E) and late (L) cropping seasons in 2015 and 2016, respectively

Table S4 LTG of HJX74 and 24 SSSLs at 15°C in four cropping seasons

SSSL code	2015E		2015L		2016E		2016L	
	LTG (%)	*P*-value	LTG (%)	*P*-value	LTG (%)	*P*-value	LTG (%)	*P*-value
HJX74	67.7±1.4		57.4±1.3		55.3±1.4		44.1±1.2	
S1	35.7±4.1	1.62E-04	18.9±3.2	2.61E-06	40.2±1.3	ns	42.1±2.2	ns
S2	9.6±1.3	8.32E-10	67.1±1.9	ns	30.1±3.1	1.19E-04	24.4±2.2	6.37E-04
S3	82.4±1.8	ns	84.0±2.8	7.80E-06	63.7±3.5	ns	58.8±1.1	6.84E-05
S4	72.5±3.0	ns	87.9±2.6	2.07E-05	67.6±1.1	4.86E-04	59.6±3.8	ns
S5	41.4±3.6	ns	nd	nd	90.9±1.5	1.67E-06	73.8±1.2	1.53E-04
S6	98.1±2.3	2.16E-05	nd	nd	87.2±2.4	3.21E-07	77.5±1.7	4.52E-07
S7	90.8±2.8	ns	nd	nd	91.1±2.1	1.59E-08	79.3±2.5	1.93E-07
S8	47.3±0.6	7.91E-09	66.3±5.8	ns	38.6±2.3	ns	23.6±2.9	4.50E-04
S9	39.5±6.0	9.28E-04	nd	nd	39.1±2.6	ns	16.9±1.9	5.20E-06
S10	24.9±2.9	1.69E-06	nd	nd	50.3±1.3	ns	24.5±2.4	7.12E-04
S11	9.9±2.2	1.08E-09	nd	nd	35.7±2.0	ns	23.7±2.5	4.68E-04
S12	79.3±2.1	ns	87.7±2.7	2.41E-05	87.7±1.0	6.82E-11	69.0±2.7	9.23E-05
S13	nd	nd	59.7±2.7	ns	85.3±3.6	1.99E-06	70.8±1.7	2.57E-05
S14	80.3±0.5	6.18E-06	nd	nd	64.8±0.6	9.64E-04	49.8±3.0	ns
S15	87.2±3.6	ns	83.7±2.8	9.80E-06	52.4±2.0	ns	76.6±1.8	3.55E-13
S16	10.8±1.7	1.72E-09	nd	nd	34.9±4.0	ns	11.6±2.3	1.06E-07
S17	2.8±4.2	5.70E-12	9.8±5.2	2.14E-09	21.7±2.6	9.49E-07	36.5±3.1	ns
S18	68.3±3.6	ns	84.7±2.5	1.33E-04	88.5±3.9	2.78E-07	64.7±3.4	ns
S19	49.7±5.1	ns	66.7±2.4	ns	93.4±1.1	3.25E-07	72.9±2.9	2.57E-04
S20	70.0±6.8	ns	86.9±3.9	4.14E-05	87.3±4.1	7.38E-07	70.8±2.9	3.60E-05
S21	27.2±4.4	5.33E-06	61.5±1.6	ns	12.8±2.4	3.89E-09	28.5±1.9	ns
S22	87.3±2.0	ns	90.8±1.5	2.66E-08	49.3±1.7	ns	75.1±3.7	8.99E-04
S23	41.5±11.8	ns	69.1±5.6	ns	81.0±3.5	4.16E-04	76.9±1.2	5.86E-07
S24	14.6±2.1	1.32E-08	nd	nd	30.0±3.2	1.12E-04	8.7±1.3	7.20E-09

2015E, 2015L, 2016E and 2016L represent the early (E) and late (L) cropping seasons in 2015 and 2016, respectively

[a] *P* values are produced by the mean comparison of arcsin-transformed germination data between SSSLs and the recipient parent HJX74 with two tail t-test. A *P*-value greater than 0.001 was defined as not significant (ns) in this study. nd, no data; ns, non significant

Table S5 Summary of genotypic means for germination and genetic effects of markers segregating in progeny lines derived from selected recombinants for *qLTG3a* region.

Recombinant	Marker[a]	Progeny line[b]			Germination (%)[c]		
		Genotype	N	Mean ± SD	*a* (Prob.)	*d* (Prob.)	R^2
S6_R1	P8	aa	26	55.6 ± 10.6	0.10 (<0.0001)	0.03 (0.14)	0.33
		Aa	45	68.5 ± 10.3			
		AA	15	74.7 ± 8.0			
S6_R2	P13	aa	10	40.0 ± 14.9	Homozygous for HJX74 allele at *qLTG3a*		ns
		Aa	47	41.7 ± 12.3			
		AA	24	42.6 ± 12.4			
S6_R3	P8	aa	17	46.3 ± 9.0	0.14 (<0.0001)	0.05 (0.02)	0.53
		Aa	44	65.0 ± 11.5			
		AA	24	73.8 ± 7.5			
S6_R4	P1	aa	20	57.9 ± 17.6	Homozygous for HJX74 allele at *qLTG3a*		ns
		Aa	45	55.9 ± 12.4			
		AA	15	60.6 ± 15.3			
S6_R5	P13	aa	15	36.7 ± 14.7	Homozygous for HJX74 allele at *qLTG3a*		ns
		Aa	35	37.5 ± 12.9			
		AA	20	40.9 ± 13.4			

[a] Markers were used for genotyping the progeny populations and evaluated for genotypic means and genetic effects.

[b] Letters indicate the marker alleles from the parental line HJX74 (a) or S6 (A), and N is the sample size.

[c] The additive effects (*a*), dominance effects (*d*), and the proportion of the variance explained by the QTL (R^2) were estimated based on Model 1. A positive (negative) *a* or *d* value indicates that the A (a) allele enhances LTG. ns, non significant

Table S6 The RAP ID, MSU ID and R498 (Shuhui498) ID of candidate genes

ORF No.	Gene RAP ID	MSU ID	R498 ID
ORF1	Os03g0213300	LOC_Os03g11460	OsR498G0305449100.01.T03
ORF2	Os03g0213400	LOC_Os03g11470	OsR498G0305449400.01.T01
ORF3	Os03g0213500	LOC_Os03g11480	OsR498G0305449700.01.T02
ORF4	Os03g0213600	LOC_Os03g11490	OsR498G0305449900.01.T04
ORF5	Os03g0213700	LOC_Os03g11500	OsR498G0305450200.01.T01
ORF6	Os03g0213800	LOC_Os03g11510	OsR498G0305450400.01.T01
ORF7	Os03g0213900	LOC_Os03g11520	OsR498G0305450900.01.T02
ORF8	Os03g0214000	LOC_Os03g11530	OsR498G0305451100.01.T03
ORF9	Os03g0214050	NA	NA
ORF10	Os03g0214100	LOC_Os03g11540	OsR498G0305451300.01.T01
ORF11	Os03g0214200	LOC_Os03g11550	OsR498G0305451600.01.T02
ORF12	Os03g0214400	LOC_Os03g11560	OsR498G0305452200.01.T03
ORF13	Os03g0214600	LOC_Os03g11570	OsR498G0305452300.01.T01
ORF14	Os03g0214900	LOC_Os03g11580	OsR498G0305452600.01.T01
ORF15	Os03g0215000	LOC_Os03g11590	OsR498G0305452800.01.T01
ORF16	Os03g0215200	LOC_Os03g11600.	OsR498G0305454900.01.T01
ORF17	Os03g0215400	LOC_Os03g11614	OsR498G0305455800.01.T03
ORF18	Os03g0215600	LOC_Os03g11630	NA
ORF19	Os03g0215700	LOC_Os03g11650	OsR498G0305456700.01.T03
ORF20	Os03g0215800	LOC_Os03g11660	OsR498G0305457100.01.T03
ORF21	Os03g0215900	LOC_Os03g11670	OsR498G0305457400.01.T01
ORF22	Os03g0216000	LOC_Os03g11680	OsR498G0305457800.01.T01
ORF23	Os03g0216300	LOC_Os03g11690	OsR498G0305459100.01.T03
ORF24	Os03g0216400	LOC_Os03g11700	NA
ORF25	Os03g0216500	LOC_Os03g11710	OsR498G0305459600.01.T01
ORF26	Os03g0216600	LOC_Os03g11720	OsR498G0305460300.01.T02
ORF27	Os03g0216700	LOC_Os03g11734	OsR498G0305460600.01.T01
ORF28	Os03g0216733	NA	NA
ORF29	Os03g0216766	NA	NA
ORF30	Os03g0216800	LOC_Os03g11760	OsR498G0305461700.01.T05
ORF31	Os03g0216900	LOC_Os03g11770	OsR498G0305462100.01.T02
ORF32	Os03g0217000	LOC_Os03g11780	OsR498G0305462300.01.T02
ORF33	Os03g0217200	LOC_Os03g11790	OsR498G0305462500.01.T03
ORF34	Os03g0217400	NA	NA
ORF35	Os03g0217801	NA	NA
ORF36	Os03g0217900	LOC_Os03g11840	NA
ORF37	Os03g0218100	LOC_Os03g11860	NA
ORF38	Os03g0218200	LOC_Os03g11874	OsR498G0305463200.01.T04
ORF39	Os03g0218300	LOC_Os03g11890	OsR498G0305463500.01.T02
ORF40	Os03g0218400	LOC_Os03g11900	OsR498G0305463800.01.T01

NA: not applicable

Table S7 Nonsynonymous SNP variations (ΔSNP-index > 0.8) of candidate genes in the *qLTG3a* locus

Chr.	start	end	ref	mut	variation type	ΔSNP-index	structure type	R498 gene ID
3	6036758	6036758	G	C	SNP	0.875	exonic	OsR498G0305449100.01
3	6038282	6038282	G	A	SNP	0.875	exonic	OsR498G0305449100.01
3	6034463	6034463	G	C	SNP	0.8462	exonic	OsR498G0305449100.01
3	6076067	6076067	T	G	SNP	1	exonic	OsR498G0305449900.01
3	6074494	6074494	C	T	SNP	0.8824	exonic	OsR498G0305449900.01
3	6071013	6071013	A	G	SNP	0.8571	exonic	OsR498G0305449900.01
3	6094873	6094873	T	C	SNP	0.8571	exonic	OsR498G0305450900.01
3	6095124	6095124	G	A	SNP	0.8535	exonic	OsR498G0305450900.01
3	6093745	6093745	G	T	SNP	0.8333	exonic	OsR498G0305450900.01
3	6104000	6104000	G	T	SNP	0.8	exonic	OsR498G0305451300.01
3	6131044	6131044	T	C	SNP	0.9	exonic	OsR498G0305452600.01
3	6131780	6131780	G	C	SNP	0.8125	exonic	OsR498G0305452600.01
3	6216063	6216063	G	A	SNP	0.9	exonic	OsR498G0305456700.01
3	6254147	6254147	G	T	SNP	0.8889	exonic	OsR498G0305459600.01
3	6285033	6285033	T	G	SNP	1	exonic	OsR498G0305461700.01
3	6295769	6295769	A	G	SNP	0.9231	exonic	OsR498G0305462300.01
3	6335221	6335221	C	A	SNP	0.9412	exonic	OsR498G0305463500.01
3	6333667	6333667	T	C	SNP	0.9091	exonic	OsR498G0305463500.01
3	6337584	6337584	T	C	SNP	0.8398	exonic	OsR498G0305463500.01

The reference genome for BSA sequencing is the indica variety R498 (Shuhui498)

Table S8 Indel variations (ΔInDel-index > 0.8) in the upstream and exonic regions of candidate genes in the *qLTG3a* locus

Chr.	start	end	ref	mut	variation type	ΔInDel-index	structure type	R498 gene ID
3	6119988	6119988	-	CCGCCTACAA	Insertion	0.80	upstream	OsR498G0305452200.01
3	6206816	6206816	-	CGAGC	Insertion	0.80	upstream	OsR498G0305456700.01
3	6206823	6206824	AA	-	Deletion	0.80	upstream	OsR498G0305456700.01
3	6068043	6068043	T	-	Deletion	0.81	upstream	OsR498G0305449900.01
3	6089138	6089138	-	T	Insertion	0.81	upstream	OsR498G0305450900.01
3	6283585	6283585	-	TC	Insertion	0.81	upstream	OsR498G0305461700.01
3	6120089	6120089	-	C	Insertion	0.82	upstream	OsR498G0305452200.01
3	6067869	6067869	-	CG	Insertion	0.83	upstream	OsR498G0305449900.01
3	6217105	6217105	-	T	Insertion	0.83	upstream	OsR498G0305457100.01
3	6078335	6078338	ACAA	-	Deletion	0.84	exonic	OsR498G0305450200.01
3	6134303	6134303	-	TATAATATATA	Insertion	0.85	upstream	OsR498G0305452600.01
3	6088700	6088702	AAC	-	Deletion	0.87	upstream	OsR498G0305450900.01
3	6120911	6120911	-	T	Insertion	0.88	upstream	OsR498G0305452300.01
3	6067561	6067561	C	-	Deletion	0.89	upstream	OsR498G0305449900.01
3	6247040	6247040	-	T	Insertion	0.89	upstream	OsR498G0305459100.01

Table S8 (continued)

Chr.	start	end	ref	mut	variation type	ΔInDel-index	structure type	R498 gene ID
3	6087497	6087523	GCCGCCAAC	-	Deletion	0.90	upstream	OsR498G0305450400.01
3	6133582	6133582	-	T	Insertion	0.92	upstream	OsR498G0305452600.01
3	6191922	6191922	-	G	Insertion	0.92	upstream	OsR498G0305455800.01
3	6095960	6095960	-	GGC	Insertion	0.95	exonic	OsR498G0305451100.01
3	6030020	6030020	-	TTTTGGTGTT	Insertion	1.00	upstream	OsR498G0305449100.01
3	6087005	6087005	-	GCTCGGT	Insertion	1.00	upstream	OsR498G0305450400.01
3	6088763	6088763	A	-	Deletion	1.00	upstream	OsR498G0305450900.01
3	6119511	6119511	-	CTGCTGGCTG	Insertion	1.00	upstream	OsR498G0305452200.01
3	6119900	6119900	T	-	Deletion	1.00	upstream	OsR498G0305452200.01
3	6120881	6120881	G	-	Deletion	1.00	upstream	OsR498G0305452300.01
3	6133735	6133735	-	A	Insertion	1.00	upstream	OsR498G0305452600.01
3	6134088	6134088	T	-	Deletion	1.00	upstream	OsR498G0305452600.01
3	6207402	6207402	-	A	Insertion	1.00	upstream	OsR498G0305456700.01
3	6274675	6274675	-	CG	Insertion	1.00	exonic	OsR498G0305460600.01
3	6330983	6330983	-	T	Insertion	1.00	upstream	OsR498G0305463200.01

The reference genome for BSA sequencing is the indica variety R498 (Shuhui498)
